# Hirano body expression impairs spatial working memory in a novel mouse model

**DOI:** 10.1186/s40478-014-0131-9

**Published:** 2014-09-02

**Authors:** Matthew Furgerson, Jason K. Clark, Jonathon D. Crystal, John J. Wagner, Marcus Fechheimer, Ruth Furukawa

**Affiliations:** 1 Department of Cellular Biology, University of Georgia, Athens, 30602 GA USA; 2 Department of Biochemistry and Molecular Biology, University of Georgia, Athens, 30602 GA USA; 3 Department of Physiology and Pharmacology, University of Georgia, Athens, 30602 GA USA; 4 Department of Psychological and Brain Sciences, Indiana University, Bloomington, 47405 IN USA

**Keywords:** Hirano bodies, Alzheimer's disease, Radial arm maze, Electrophysiology, Open field test, Transgenic mouse model, Inflammation, Neurodegeneration, Hippocampal slice

## Abstract

**Introduction:**

Hirano bodies are actin-rich intracellular inclusions found in the brains of patients with neurodegenerative conditions such as Alzheimer's disease or frontotemporal lobar degeneration-tau. While Hirano body ultrastructure and protein composition have been well studied, little is known about the physiological function of Hirano bodies in an animal model system.

**Results:**

Utilizing a Cre/Lox system, we have generated a new mouse model which develops an age-dependent increase in the number of model Hirano bodies present in both the CA1 region of the hippocampus and frontal cortex. These mice develop normally and experience no overt neuron loss. Mice presenting model Hirano bodies have no abnormal anxiety or locomotor activity as measured by the open field test. However, mice with model Hirano bodies develop age-dependent impairments in spatial working memory performance assessed using a delayed win-shift task in an 8-arm radial maze. Synaptic transmission, short-term plasticity, and long-term plasticity was measured in the CA1 region from slices obtained from both the ventral and dorsal hippocampus in the same mice whose spatial working memory was assessed. Baseline synaptic responses, paired pulse stimulation and long-term potentiation measurements in the ventral hippocampus were indistinguishable from control mice. In contrast, in the dorsal hippocampus, synaptic transmission at higher stimulus intensities were suppressed in 3 month old mice with Hirano bodies as compared with control mice. In addition, long-term potentiation was enhanced in the dorsal hippocampus of 8 month old mice with Hirano bodies, concurrent with observed impairment of spatial working memory. Finally, an inflammatory response was observed at 8 months of age in mice with Hirano bodies as assessed by the presence of reactive astrocytes.

**Conclusion:**

This study shows that the presence of model Hirano bodies initiates an inflammatory response, alters hippocampal synaptic responses, and impairs spatial working memory in an age-dependent manner. This suggests that Hirano bodies may promote disease progression. This new model mouse provides a tool to investigate how Hirano bodies interact with other pathologies associated with Alzheimer's disease. Hirano bodies likely play a complex and region specific role in the brain during neurodegenerative disease progression.

**Electronic supplementary material:**

The online version of this article (doi:10.1186/s40478-014-0131-9) contains supplementary material, which is available to authorized users.

## Introduction

Neurodegenerative diseases are characterized by neuronal loss which results in progressive cognitive decline, motor impairments, and changes in behavior [1]. The most prevalent neurodegenerative disease is Alzheimer's disease [2],[3] which is characterized pathologically by the deposition of protein aggregates [1]. The brains of Alzheimer's disease patients develop extracellular amyloid beta plaques and intracellular neurofibrillary tangles (NFTs) [4],[5]. In addition, patients with Alzheimer's disease and other neurodegenerative diseases may also develop a secondary pathology known as Hirano bodies [6]–[10].

Hirano bodies are intracellular, eosinophilic rod-shaped inclusions found in both neurons and glia of the central nervous system [6]–[12]. Hirano bodies are paracrystalline structures primarily composed of filamentous actin (F-actin) and actin-associated proteins [7],[13]. Hirano bodies are differentiated from other types of actin inclusions based on their ultrastructure [14]. They have a distinct orientation and spacing of F-actin, the appearance of which changes based on the plane of section. These filaments are approximately 6–10 nm wide with 10–12 nm spacing between parallel filaments [15],[7],[16]. In addition to actin-associated proteins, amyloid precursor protein intracellular domain (AICD) and tau are present in Hirano bodies, implicating these structures in the pathogenesis of Alzheimer's disease [17]–[19].

Despite decades of research, very little is known about the physiological role of Hirano bodies. Due to a lack of a mammalian model system, research has been limited to studying Hirano body frequency, components, and structure in post-mortem tissue. However, the development of a model system for studying Hirano bodies was made possible through expression of a c-terminal 34 kDa actin-binding protein truncation mutant (CT, amino acids 124–295) in *Dictyostelium discoideum*[20],[21]. Expression of CT does not affect total actin levels, but causes redistribution in the ratio of globular to F-actin [21]. These structures slow *Dictyostelium* growth and development only moderately, and are not detrimental to cell survival [21]. Ultrastructural analysis of CT-induced actin-rich deposits revealed highly ordered actin filaments identical to Hirano bodies from human tissue [21]. Formation of model Hirano bodies through expression of CT has also been successful in immortal mammalian cell lines as well as primary neurons [22]. Cells expressing CT experience no growth or migration phenotypes [22]. Model Hirano bodies formed in mammalian cells contain many of the same protein components as Hirano bodies found in humans as well as the same hallmark ultrastructure and filament spacing [22]–[24].

In addition to structural characterization of model Hirano bodies, limited physiological aspects of these inclusions has also been investigated. Both Hirano bodies found in humans and model Hirano bodies are often seen enclosed in membranes thought to be derived from autophagosomes [12],[22]. Consistent with this hypothesis, model Hirano bodies are degraded through both the autophagy and proteasome pathways determined through use of pharmacology and an autophagy mutant *Dictyostelium*[25]. The impact of model Hirano bodies on AICD and tau was investigated in cell culture since these proteins are colocalized with both human and model Hirano bodies [19],[24],[23],[22]. Normally AICD is abundant in the nucleus, where it plays a role in transcription [26],[27]. The presence of model Hirano bodies drastically reduces AICD nuclear localization [23]. The presence of model Hirano bodies was also found to decrease AICD- and tau-induced cell death and AICD-induced transcription [23],[24]. These results imply both a potentially protective and deleterious role for Hirano bodies.

While cell culture is useful for mechanistic characterization at the cellular level, an *in vivo* model is needed to provide the appropriate anatomical and physiological context for an informative assessment of systemic pathology. Therefore, a mouse with brain specific expression of CT fused to green fluorescent protein under control of the Rosa promoter and induced by Cre/lox technology was generated by crossing a CT-GFP transgenic mouse (R26CT) with a Thy1.2-CRE mouse [28]. These mice developed rod-shaped eosinophilic inclusions primarily in the CA3 region of the hippocampus. In addition, these inclusions had an ultrastructure indistinguishable from Hirano bodies found in humans [28]. The formation of model Hirano bodies in the mouse hippocampus did not induce neuronal loss or impact long-term plasticity. However, a deficit in presynaptic short-term plasticity in the CA1 region was observed [28]. Despite these interesting findings, complex behavioral studies such as motor or learning tasks were not evaluated. Furthermore, Hirano bodies in human disease are found predominately in the CA1 region of the hippocampus, not the CA3 as seen in the previous mouse model [12],[16]. In order to generate Hirano bodies in the CA1 region of the hippocampus, R26CT mice were crossed with a CamKIIa-CRE mouse, which directs CRE expression predominately to the CA1 region of the hippocampus and forebrain [29],[30]. In the current study, this new mouse model is characterized utilizing electrophysiological, pathological, and behavioral methods to evaluate the impact of model Hirano bodies on neurophysiology and cognition.

## Materials and methods

### Animals

*C57Bl/6-Gt(ROSA)26Sor*^*tm1(CT-GFP)UGA*^ (R26CT) mice (previously described [28]) were crossed with CamKIIa-CRE mice (*B6.Cg-Tg(Camk2a-cre)T29-1Stl/J*, Jax ID: 005359) [29] to induce expression of CT-GFP (R26CT-CRE). During breeding, CRE was carried maternally and all mice were homozygous for the CT-GFP transgene. All R26CT and R26CT-CRE mice used in behavioral and electrophysiological studies were male.

PCR was utilized to genotype the presence of R26CT with primers: P1 5′ -TTGGAGGCAGGAAGCACTTG -3′; P2 5′ -CATCAAGGAAACCC TGGACTACTG- 3′; and P3 5′ -CCGACAAAACC GAAAATCTGTG-3′ using genomic DNA obtained from the tail as a template. Amplification using P1 and P2 yields a 230 bp product from the R26CT allele and P1 and P3 yields a 369 bp product from the wild type Rosa26 allele. To detect Cre transgene utilizing PCR, the primers were: 5′ -CCAGGCCTTTTCTGAGCATACC- 3′ and 5′ -CAACACCATTTTTTCTGACCCG-3′, producing a product of 641 bp.

Mice had ad libitum access to food (except during behavioral studies, see below) and water during this study. All animal protocols and experiments were approved by the University of Georgia Institutional Animal Care and Usage Committee.

### Statistics

Tests of significance were performed using both paired and unpaired t-test, and mixed ANOVA. Results were considered significant if p < 0.05.

### Brain sectioning and histology

For cryosections utilized in immunofluorescence, dissected whole brains were fixed in 4% paraformaldehyde in phosphate buffered saline, pH 7.4 (PBS) overnight, followed by cryoprotection in 30% sucrose, embedding in OCT (Optical Cutting Temperature, Tissue-Tek 4583), and storage in liquid nitrogen. 6–8 μm thick sagittal sections were cut from frozen tissue using a cryostat (Leica CM3050 S, Richmond, IL) and electrostatically attached to Superfrost Plus glass slides (Fisher Scientific, Pittsburgh, PA). For paraffin sections, dissected brains were fixed in 4% paraformaldehyde in PBS, pH 7.4, at 4°C overnight, dehydrated in a graded series of 50, 75, 90, 96 and 100% ethanol, equilibrated with xylene, embedded in paraffin, and sectioned on a sliding microtome (Leica RM2155, Richmond, IL) at a thickness of 5–8 μm and mounted on slides. H&E staining was performed as follows: after dewaxing with xylene, sections were stained with Gill's No. 2 hematoxylin (Sigma-Aldrich Chemical Co., St. Louis, MO) and eosin solution (Sigma-Aldrich Chemical Co., St. Louis, MO).

### Immunohistochemistry

Mounted paraffin sections were dewaxed in xylene and rehydrated in graded ethanol solutions prior to antigen retrieval in boiling 50 mM sodium citrate plus 0.01% Tween20 for 25 minutes. Endogenous peroxidase activity was inhibited through incubation of sections in 3% hydrogen peroxide for 10 minutes prior to washing with PBS and blocking with 10 mg/ml bovine serum albumin (BSA) in PBS overnight. Slices were incubated in mouse anti-GFAP (1/1000) (Sigma-Aldrich Chemical Co., St. Louis, MO) or mouse anti-ED1 (1/400) (Abcam, Cambridge, MA) primary antibodies for 1 hour. Secondary biotinylated goat anti-mouse and goat anti-rabbit antibodies were used at 1/450 dilution for 1 hour. Slices were incubated with streptavidin-HRP polymer complex (1/1000) (Vector Laboratory, Burlingame, CA) for 30 minutes. Slices were washed 3 times for 5 minutes each between antibody and enzyme incubations with TBST (10 mM Tris–HCl, pH 7.4, 150 mM NaCl, 0.1% Tween20). Diaminobenzidine (DAB) enhanced substrate system was used according to the manufacturer's instructions (Vector Laboratory, Burlingame, CA). After washing off excess DAB substrate, slides were counterstained with Gill's No. 2 Hematoxylin (Sigma-Aldrich Chemical Co., St. Louis, MO) prior to mounting. Sections were viewed with a Leica DM6000 B microscope (Wetzlar, Germany) with Hamamatsu ORCA-ER digital camera (Hamamatsu, Bridgewater, NJ).

### Immunofluorescence

Cryosections were blocked for 1 hour in 2% BSA in TBST and incubated in primary antibody at room temperature overnight. The sections were washed three times in 4% milk in TBST for 5 min each, followed by 1 hour incubation with rabbit anti-GFP (1/500) (Sigma-Aldrich Chemical Co., St. Louis, MO), FITC-labeled goat anti-rabbit secondary (1/1000) (Sigma-Aldrich Chemical Co., St. Louis, MO), TRITC-conjugated phalloidin (1/40) (Sigma-Aldrich Chemical Co., St. Louis, MO), and 264 μM Hoechst 33258 with appropriate washes in between. Slides were visualized with a Zeiss Axioobserver Z1 equipped with an AxioCam MRm controlled by AxioVision4.6 software.

### Transmission electron microscopy (TEM)

TEM was performed as previously described with slight modification [28]. Whole mouse brains were dissected to separate hippocampus from cortex. Hippocampal tissue blocks were fixed by immersion in 4% paraformaldehyde and 2% glutaraldehyde in 0.1 M cacodylate buffer, pH 7.4 overnight, and postfixed in 1% osmium tetroxide for 2 hours. After serial dehydration in ethanol solutions, tissues were embedded in Epon (Embed-812; Electron Microscope Science, Hatfield, PA). Semithin sections were stained with 1% toluidine blue in 1% sodium tetraborate. Ultrathin sections were collected on nickel grids, counterstained with uranyl acetate for 30 minutes, and followed by lead citrate for 5 minutes at room temperature. Samples were observed with a JEOL 100CX with an accelerating voltage of 80 kV.

### Western blot

Brain samples were dissected from mice, flash frozen in liquid nitrogen, and stored at −80°C until processed. Tissues were homogenized in a Potter-Elvehjem homogenizer containing 4 brain volumes of Tris buffered saline (25 mM Tris–HCl pH 7.4, 140 mM NaCl, 3 mM KCl, 5 mM EDTA, and 2 mM 1,10-phenanthroline) with 10 μL protease inhibitor cocktail (5 mM EGTA, 1 mM DTT, 100 mM leupeptin, 10 mM pepstatin, 0.1 M PMSF, 0.1 M benzamidine, and 0.5 M ε-aminocaproic acid). Cell debris was separated from total homogenate by centrifugation at 13,000 g for 15 min at 4°C. Supernatant was stored at −80°C until used. Protein concentrations of the supernatants were determined by bicinchoninic acid assay using BSA as a standard [31]. For western blot analysis, tissue samples were loaded at equal total protein, separated by SDS-PAGE, and transferred to nitrocellulose membranes. Blots were blocked in 5% nonfat dry milk in TBST and probed using either mouse anti-GFAP (1/2000) (Sigma-Aldrich Chemical Co., St. Louis, MO), rabbit anti-GFP (1/5000) (Sigma-Aldrich Chemical Co., St. Louis, MO), mouse anti-ED1 (1/5000) (Abcam, Cambridge, MA), mouse anti-synaptophysin (1/2000) (AbD Serotec, Raleigh, NC), or mouse anti-alpha tubulin (1/8000) (Millipore, Billerica, MA). After three washes with TBST, blots were incubated with either goat anti-mouse or goat anti-rabbit HRP conjugated secondary antibodies (1/10000) (Pierce-lab, Rockford, IL) and detected by chemiluminescence using SuperSignal Western Dura Extended Duration Substrate (Thermo Scientific, Rockford IL). Images were captured utilizing ChemiDoc™ MP system and Image Lab™ software (Bio-Rad Laboratories, Hercules, CA).

### Assessment of learning and memory in the radial arm maze

Learning and memory assessments were conducted using an 8-arm radial mouse maze (Med Associates, St. Albans, VT) as similarly described for rats [32]. This maze consists of eight arms extending from a central chamber with eight guillotine doors positioned at the interface of the central chamber and arms. A 20-mm food dispenser and trough are at the end of each arm. Each arm has two sets of photosensors to track movement of mice in and out of the arms. In addition, the food trough also contains photosensors that detect mouse head entries and dispense food. The sides and top of each arm are composed of clear plastic to allow mice to use visual cues in the room to spatially navigate the maze. A computer in an adjacent room controlled the maze events and data collection. Photosensor, food, and door data were collected using MED-PC software 4.0 (Med Associates, St. Albans, VT) with a resolution of 10 ms. A video camera was mounted above the maze to visualize the mice during the procedure.

Behavioral assessment in the radial arm maze was performed at either 3 or 8 months of age. Thirteen days prior to the start of behavioral testing, mice were individually housed and a three-day average of individual body weight was determined. Mice were diet restricted to reduce and maintain a body weight of ~87.0% of their ad libitum food body weight for the duration of the behavioral assessment. For four days prior to testing, mice were pre-trained to associate the maze with the experience of obtaining a sucrose-flavored food reward (Bio-Serve F0071, Frenchtown, NJ) by allowing each animal free access to four of the eight arms until one food reward from each arm was retrieved.

The maze was cleaned between subjects with 1/1250 diluted Coverage Plus NPD disinfectant (Steris Life Sciences, Mentor, OH) to prevent a previous mouse's scent from interfering with a subsequent mouse's performance. To further prevent a mouse from using its own scent cues, the entire maze was scent saturated using cotton bedding from the mouse's home cage.

### Radial arm maze training phase (8 arms open, 8 arms baited) to assess spatial short-term memory

Each mouse was placed in the central chamber of the maze for a two minute acclimation period before beginning the procedure. After acclimation, all eight doors opened with the objective of collecting a food reward available at the end of each arm (8 arms open, 8 arms baited). Only one food reward is delivered per arm, and a revisit to a previously visited food trough is considered an error in spatial short-term memory. After either collecting the last food reward or after 15 minutes of elapsed time, the session ends and the doors close. These training phase sessions are performed once a day, at the same time of day per mouse for 10 consecutive days. Results are reported as the mean total errors ± standard error of the mean (SEM) from the first 3 days or last 3 days of training. Following 10 days of consecutive spatial memory training, animals proceeded directly to the spatial working memory test with a retention interval.

### Radial arm maze test phase (delayed spatial win-shift) to assess spatial working memory

Mice were tested using a delayed spatial win-shift task for 10 consecutive days. This is a 2-phase procedure composed of a study phase and test phase. In the study phase, animals are placed in the central chamber of the maze for a two minute acclimation. Four of the eight doors are opened (randomly chosen by the computer for each mouse every day) and the mouse must collect a food reward that is available at the end of each of the 4 baited arms (4 arms open, 4 arms baited). After collecting the last food reward (or 15 minutes of elapsed time), the doors close. The mouse is subjected to a short retention interval (time delay) by being taken out of the maze and returned to his home cage for 3 minutes, during which the maze was cleaned. The mouse is returned to the maze to begin the test phase. After 1 minute of acclimation, all eight doors of the maze open. Only arms that were previously closed in the study phase are baited in the test phase (8 arms open, 4 arms baited). A revisit to a food trough previously visited in either phase is considered an error in spatial working memory. After either collecting the last food reward or after 15 minutes of elapsed time, the session ends and the doors close. Results are reported as the mean total errors ± standard error of the mean (SEM) obtained from the first 3 days or last 3 days of the test phase.

### Open field test

#### Apparatus

Activity was measured in 43.2 × 43.2 cm square chambers with clear plastic walls and a smooth metal floor (Med Associates, St. Albans, VT, USA). The chambers are individually housed in sound attenuating cubicles with a 20 lux bulb in each of the two rear corners and a ventilation fan. Two strips containing 16 infrared photobeams perpendicular to each other, with paired photodetectors mounted across from them create a 16 × 16 photobeam grid 2 cm from the floor. Activity Monitor software counts interruptions in the photobeam to determine both ambulatory (sequential) and stereotypic (repetitive) movements based on patterning of beam interruptions.

#### Locomotor activity

Five days after completion of radial arm maze testing, each mouse was placed in the center of the open field apparatus and allowed to roam freely for 60 minutes. Both ambulatory and stereotypic counts were combined into total horizontal counts and binned into 10 minute increments. For center zone analysis, a square zone of 26.3 × 26.3 cm (37.5% of total area) in the center of the chamber was designated to count crossings into this area.

### Extracellular field recording

Hippocampal slices were prepared from 3 and 8 month old R26CT and R26CT-CRE mice 10–17 days after completion of radial arm maze testing. Mice were deeply anesthetized with halothane prior to decapitation. The brain was removed and submerged in ice-cold, oxygenated (95% O_2_/5% CO_2_) dissection artificial cerebrospinal fluid (ACSF) containing: 120 mM NaCl, 3 mM KCl, 4 mM MgCl_2_, 1 mM NaH_2_PO_4_, 26 mM NaHCO_3_, and 10 mM glucose. The brain was sectioned into 400 μm thick horizontal slices using a vibratome. Horizontal and coronal sections were prepared from either half of the brain to obtain both the ventral (vH) and dorsal (dH) hippocampal slices, respectively, from the same mice. The hippocampus was sub-dissected out and a majority of the CA3 portion removed. Slices were placed in a submersion recording chamber and perfused at approximately 1 ml/min with oxygenated (95% O_2_/5% CO_2_) standard ACSF containing: 120 mM NaCl, 3 mM KCl, 1.5 mM MgCl_2_, 1 mM NaH_2_PO_4_, 2.5 mM CaCl_2_, 26 mM NaHCO_3_, and 10 mM glucose at room temperature. Slices recovered for 45 minutes at room temperature and an additional 45 minutes at 30°C. All recordings were obtained under continuous perfusion of oxygenated ACSF at 30°C. A bipolar stimulating electrode (Kopf Instruments, Tujunga, CA) was placed within the stratum radiatum of CA1 and an extracellular recording microelectrode (1.0 MΩ tungsten recording microelectrode, World Precision Instruments, Sarasota, FL) was positioned in the same layer. Field excitatory post-synaptic potentials (fEPSPs) were recorded at CA3-CA1 synapses using a stimulus pulse consisting of a single square wave of 270 μs duration. Data were digitized at 10 kHz, low-pass filtered at 1 kHz, and analyzed with pCLAMP 10.2 software (Axon Instruments, Sunnyvale, CA). The initial slope of the population fEPSP was measured by fitting a straight line to a 1 ms window immediately following the fiber volley. Stimulus response curves were obtained at the beginning of each experiment with stimulus pulses delivered at 30, 40, 50, 60, 75, 90, 110, 130, 150, and 170 μA once every 60 s (0.0167 Hz). To begin baseline recording, the stimulation intensity was adjusted to obtain a fEPSP of approximately 35-40% of the linear range between the minimum and maximum response. Paired-pulse responses were performed at intervals of 50, 100, 200, and 500 ms. The slope of paired-pulse responses was measured from an average of five pairs of pulses for each interval. Synaptic responses for long-term potentiation (LTP) experiments were normalized by dividing all fEPSP slope values by the average of the five responses recorded during the 5 minutes immediately prior to high frequency stimulation (HFS). The HFS protocol used to induce LTP in all experiments consisted of three episodes of 100 Hz stimulus trains (100 pulses) for 1 s administered at 20 s inter-train intervals. LTP values for the 1, 2, and 3 hour time points were determined by averaging 5 minutes of normalized slope values immediately prior to the 60, 120, and 180 min time marks post-HFS, respectively. Reported n-values (x(y)) indicate the number of slices (x) and the number of mice (y) assessed.

## Results

### Mouse characterization

Mice expressing CT-GFP in the hippocampus and frontal cortex were generated by crossing R26CT mice (as described previously [28],[29]) to CamKIIa-CRE mice. To verify that CT-GFP was expressed, immunofluorescence was performed on cryosections of 1 month old R26CT and R26CT-CRE mice using anti-GFP antibodies (Additional file 1: Figure S1). The presence of CT-GFP was also verified by western blot analysis using tissue from cerebral cortex and hippocampus of R26CT and R26CT-CRE mice (Additional file 1: Figure S1).

**Figure 1:**
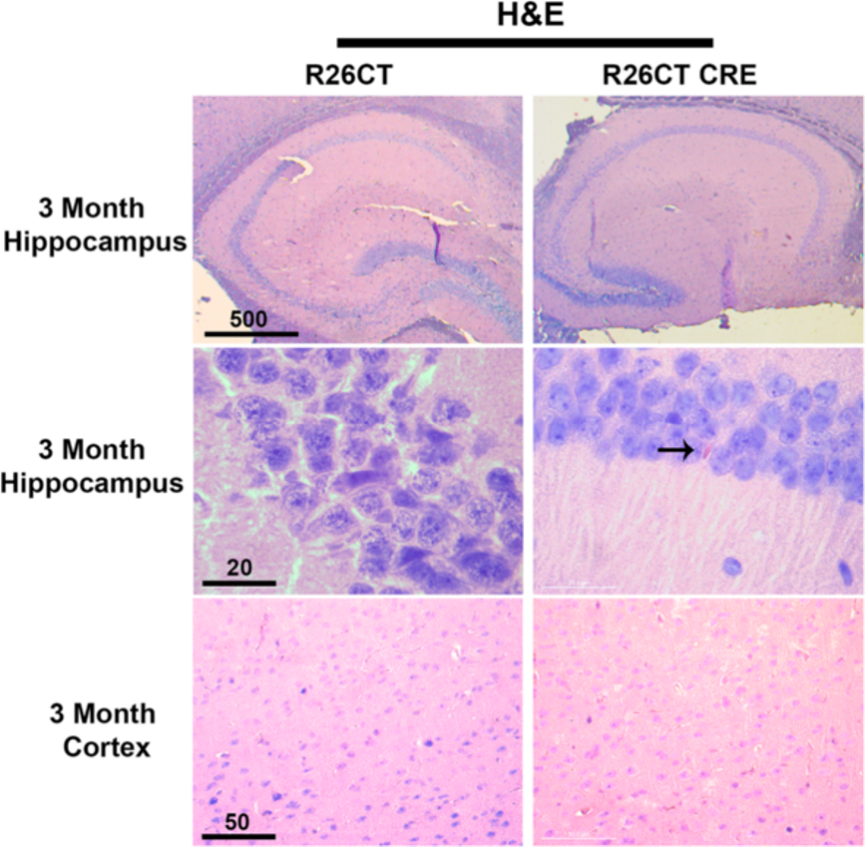
**Model Hirano bodies detected as eosinophilic inclusions in 3 month old R26CT-CRE mice.** Paraffin embedded brain sections from 3 month old R26CT and R26CT-CRE mice were dewaxed and stained with Gill's hematoxylin and counterstained with eosin. 3 month old R26CT mice show no rod-shaped eosinophilic inclusions in the pyramidal cells of the hippocampus or cerebral cortex. 3 month old R26CT-CRE mice show no inclusions in the cerebral cortex, but contain rare eosinophilic inclusions in CA1 pyramidal cells of the hippocampus indicated by the arrow. Scale bars represent 20, 50, or 500 μm.

**Figure 2:**
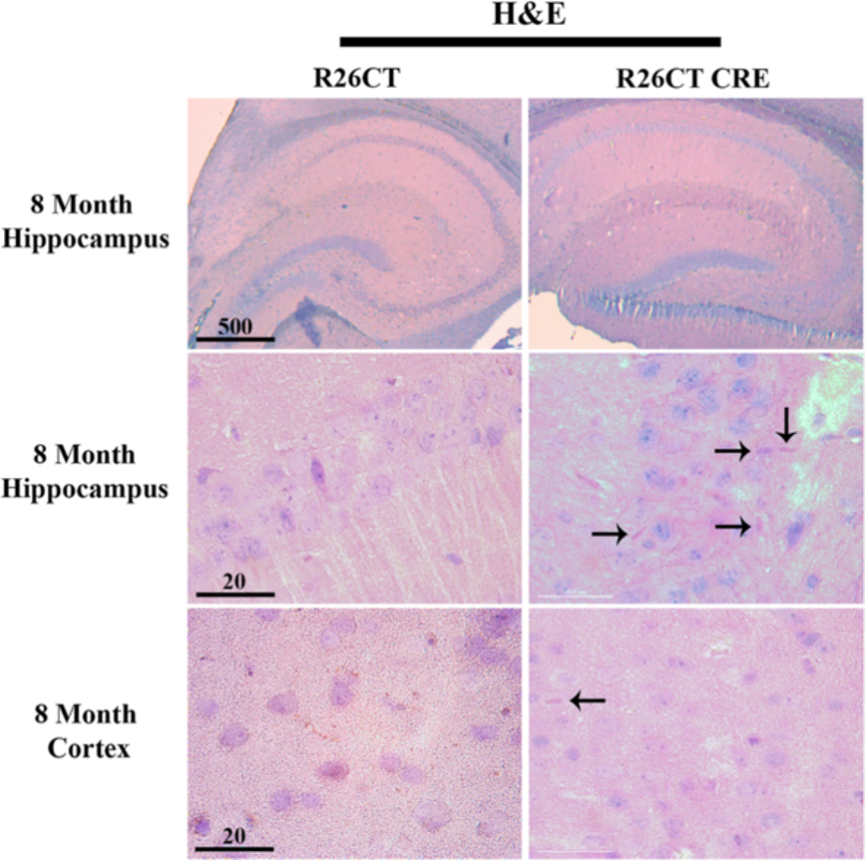
**Model Hirano bodies detected as eosinophilic inclusions in 8 month old R26CT-CRE mice.** Paraffin embedded brain sections from 8 month old R26CT and R26CT-CRE mice were dewaxed and stained with Gill's hematoxylin and counterstained with eosin. 8 month old R26CT mice show no rod-shaped eosinophilic inclusions in the pyramidal cells of the hippocampus or cerebral cortex. R26CT-CRE mice have eosinophilic inclusions predominantly in the CA1 pyramidal cell layer of the hippocampus and rarely in the cerebral cortex. Arrows indicate inclusions. Scale bars represent 20 or 500 μm.

To determine if expression of CT-GFP resulted in the production of Hirano bodies, hematoxylin and eosin staining was performed on paraffin sections of brains from 3 and 8 month old R26CT and R26CT-CRE mice. No eosinophilic inclusions were observed in 3 month old R26CT mice in either the cortex or the hippocampus (dorsal and ventral) (Figure 1). At low frequency, 3 month old R26CT-CRE mice exhibited eosinophilic inclusions in the CA1 region of the hippocampus, but not in the cortex (Figure 1). In 8 month old R26CT-CRE mice, eosinophilic inclusions appear predominantly in the CA1 of the hippocampus (dorsal and ventral) and are found rarely in the pre-frontal cortex (Figure 2). To verify that the eosinophilic inclusions found in the brains of R26CT-CRE animals have the same ultrastructure as Hirano bodies found in human brains, hippocampal samples from 8 month old mice were processed and viewed using transmission electron microscopy. R26CT-CRE mice show electron dense inclusions with 10–12 nm spacing similar to human Hirano bodies (Additional file 2: Figure S2). Structures with the characteristic ultrastructural features of Hirano bodies were not observed in samples from R26CT control mice.

**Figure 3:**
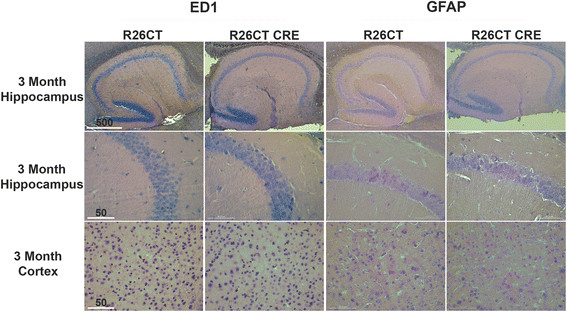
**Lack of inflammation in microglia and astrocytes of 3 month old R26CT and R26CT-CRE mice.** Paraffin embedded brain sections from 3 month old R26CT and R26CT-CRE mice were dewaxed and stained with DAB using antibodies against ED1 or GFAP to label activated microglia and reactive astrocytes, respectively. 3 month old R26CT and R26CT-CRE mice show no GFAP or ED1 staining in either the hippocampus or cortex. Scale bars represent 50 or 500 μm.

**Figure 4:**
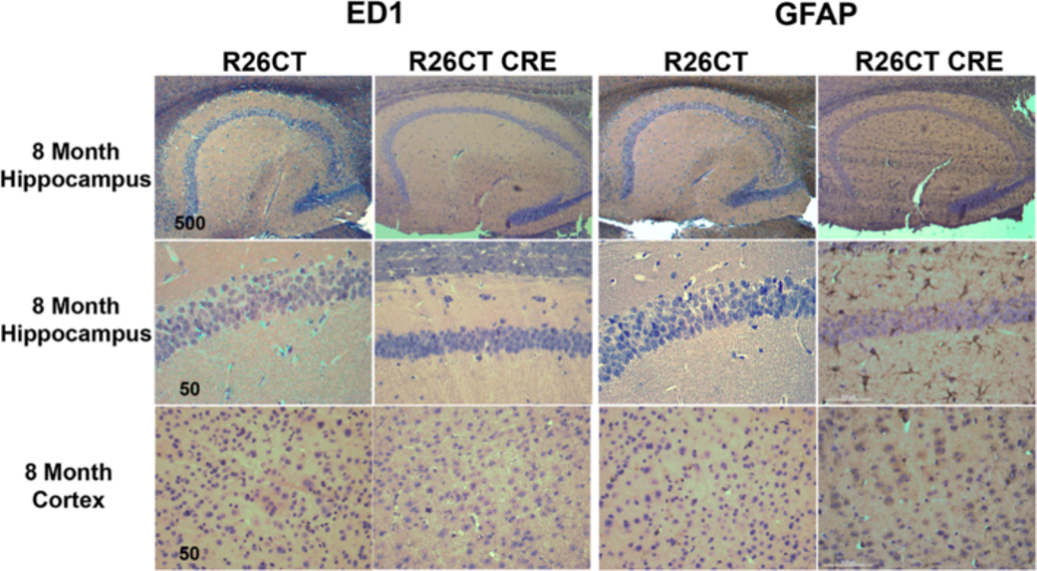
**Inflammatory response in astrocytes, but not microglia of 8 month old R26CT-CRE mice.** Paraffin embedded brain sections from 8 month old R26CT and R26CT-CRE mice were dewaxed and stained with DAB using antibodies against ED1 or GFAP to label activated microglia and reactive astrocytes, respectively. 8 month old R26CT and R26CT-CRE show no ED1 staining in the hippocampus or cerebral cortex. R26CT mice also show no GFAP staining in either hippocampus or cerebral cortex. R26CT-CRE mice have GFAP staining in the hippocampus but not cerebral cortex. Scale bars represent 50 or 500 μm.

**Figure 5:**
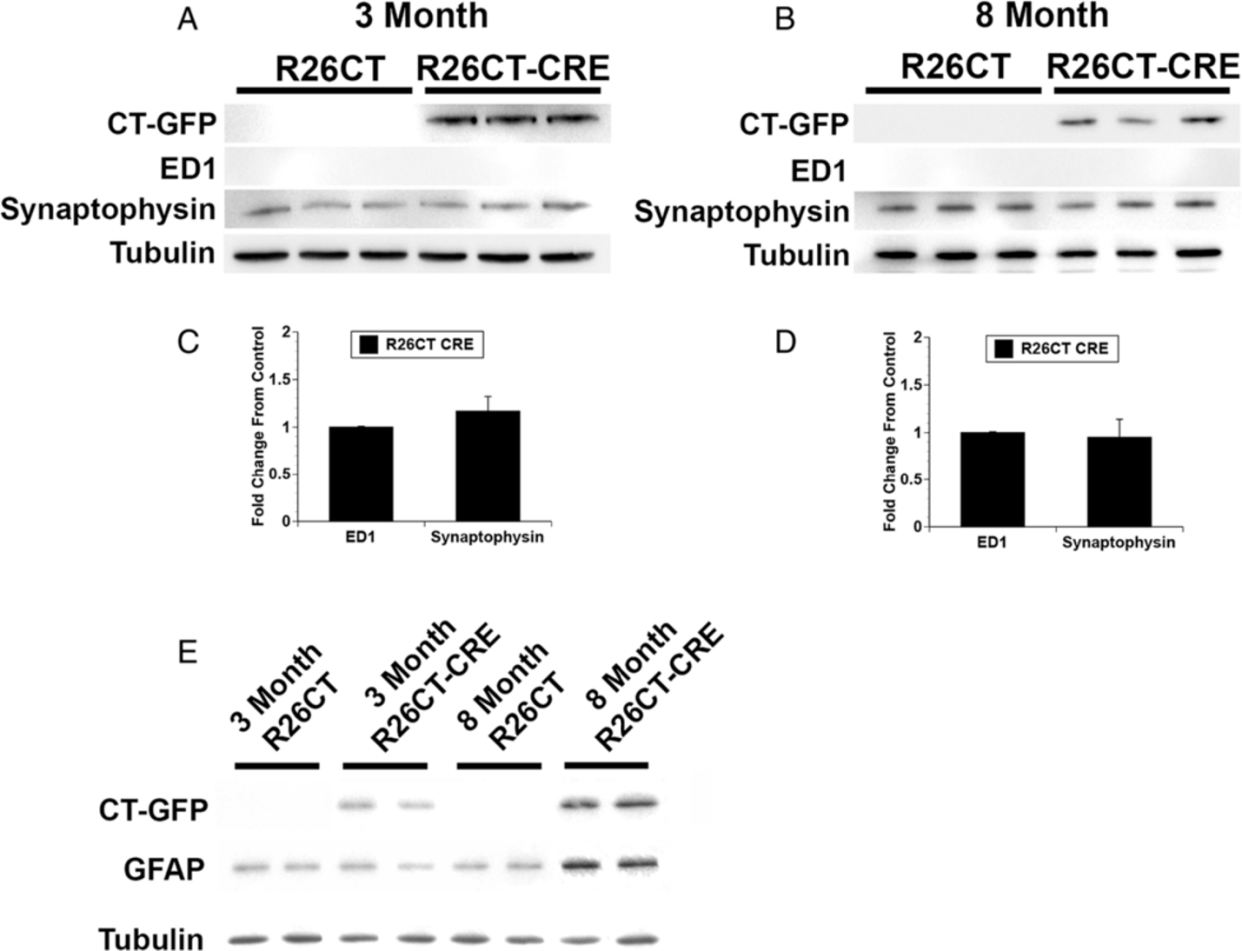
**Western blot analysis of inflammatory response in 3 and 8 month old mice.** Brain homogenate from 3 and 8 month old R26CT and R26CT-CRE mice was separated by SDS-PAGE and transferred to nitrocellulose. Blots were probed for tubulin (as a loading control), GFP, ED1, and GFAP. **A)** At 3 months of age, there is no difference in levels of synaptophysin or ED1 between R26CT and R26CT-CRE mice. **B)** At 8 months of age, there is no difference in levels of ED1 or synaptophysin between R26CT and R26CT-CRE mice. **C)** Quantification of data in **A**. **D)** Quantification of data in **B**. **E)** Levels of GFAP increased in R26CT-CRE mice at eight months of age, indicating the presence of an inflammatory response.

Since inflammation has been widely reported in neurodegenerative diseases [33], we determined whether the presence of model Hirano bodies induces an inflammatory response in the brains of 3 and 8 month old R26CT and R26CT-CRE mice. Paraffin embedded brain sections were stained with antibodies against known markers of reactive astrocytes (GFAP) and activated microglia (ED1) using DAB to visualize the product (Figures 3, 4). During inflammation, the levels of GFAP are significantly higher in reactive astrocytes [34]. Therefore, GFAP antibodies were titrated to label only reactive astrocytes using a well established 5xFAD model of Alzheimer's disease known to have inflammation (data not shown) [35]. At 3 months of age, neither R26CT nor R26CT-CRE mice show GFAP or ED1 staining in either hippocampus or frontal cortex, indicating that neither reactive astrocytes nor activated microglia are present (Figure 3). ED1 staining of 8 month old R26CT and R26CT-CRE brain sections revealed no activated microglia in either hippocampus or cortex (Figure 4). Furthermore, 8 month old R26CT mice show no GFAP staining in either hippocampus or cortex. However, 8 month old R26CT-CRE mice have GFAP staining in the hippocampus but not the cortex, indicating that the presence of model Hirano bodies induces inflammation at a later age (Figure 4). To verify these results, western blot analysis was performed using brain homogenate from 3 and 8 month old R26CT and R26CT-CRE mice (Figure 5). At 3 months of age, neither ED1 nor GFAP levels were significantly different between R26CT and R26CT-CRE mice (Figure 5). At 8 months of age, ED1 levels were not different between R26CT and R26CT-CRE mice (Figure 5A,B). In contrast, at 8 months of age, GFAP levels were increased in R26CT-CRE mice compared to R26CT mice (Figure 5C,D). These results are consistent with the immunohistochemistry results indicating that older R26CT-CRE mice have inflammation in the hippocampus as indicated by positive GFAP staining in reactive astrocytes.

### Behavioral assessment

**Figure 6:**
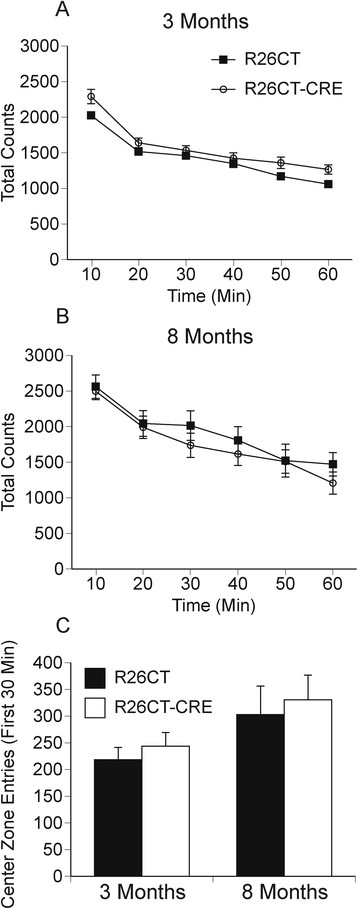
**Analysis of the open field test for 3 and 8 month old R26CT and R26CT-CRE mice.** Locomotor activity and center zone entrances of R26CT (3 month old, n = 11; 8 month old, n = 11) and R26CT-CRE (3 month old, n = 12; 8 month old, n = 12) mice were measured utilizing an open field test. **A, B)** There was no difference in locomotor activity between R26CT (black squares) and R26CT-CRE mice (open circles) at 3 or 8 months of age. **C)** R26CT (black bars) and R26CT-CRE (white bars) mice made similar entrances to the center zone. At both ages, R26CT and R26CT-CRE mice are not significantly different from each other. Error bars represent SEM.

Changes in anxiety and cognition are two possible clinical symptoms of patients suffering from neurodegenerative conditions presenting Hirano bodies [36]. Behavioral performance of R26CT and R26CT-CRE mice was assessed in an open field test and radial arm maze. In the open field test, locomotor activity and center zone entrances were evaluated in both R26CT (3 month old, n = 11; 8 month old, n = 11) and R26CT-CRE mice (3 month old, n = 12; 8 month old, n = 12). There was no difference in locomotor activity between R26CT and R26CT-CRE mice at either age (Figure 6A,B). R26CT and R26CT-CRE mice also made a similar number of entrances to the center zone, suggesting no differences in anxiety at either age (Figure 6C).

**Figure 7:**
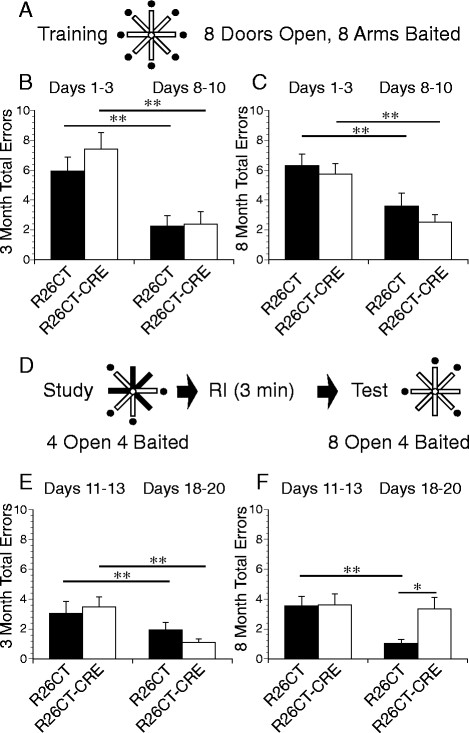
**Spatial working memory performance in the 8-arm radial maze for R26CT and R26CT-CRE mice. A)** Schematic diagram of the training phase procedure (8 arms open, 8 arms baited). **B)** At 3 months of age: R26CT (black bars, n = 11), R26CT-CRE (white bars, n = 12). **C)** At 8 months of age: R26CT (black bars, n = 11), R26CT-CRE (white bars, n = 12). No significant differences were found between R26CT and R26CT-CRE mice at either 3 or 8 months of age in the training phase **(B, C)**. Performance of both mice improved with experience. **D)** Schematic diagram of the delayed spatial win-shift assay (RI = retention interval). **E)** At 3 months of age: R26CT (black bars, n = 11), R26CT-CRE (white bars, n = 12). Both R26CT and R26CT-CRE mice improved with experience and there was no significant difference between genotypes at either days 11–13 or 18–20. **F)** At 8 months of age: R26CT (black bars, n = 11), R26CT-CRE (white bars, n = 12). R26CT mice improve with experience while R26CT-CRE mice do not. Furthermore, there is a significant difference between R26CT and R26CT-CRE mice during the late time block (days 18–20) indicating that the spatial working memory of R26CT-CRE mice is impaired. Bars represent the mean total error ± SEM of the first 3 days and the last 3 days of either the training or test phase performance. Significance between performance blocks and between genotypes was determined using a mixed ANOVA analysis (*p < 0.05, ** p < 0.01).

Spatial memory performance of R26CT and R26CT-CRE mice was evaluated at both 3 and 8 months of age utilizing a radial arm maze. The training phase (8 arms open, 8 arms baited) consisted of collecting a food reward at the end of each of the 8 arms without revisiting any previously visited locations as described in materials and methods (Figure 7A). R26CT (3 month old, n = 11; 8 month old, n = 11) and R26CT-CRE mice (3 month old, n = 12; 8 month old, n = 12) performed similarly in this task, as both groups showed significant improvement in performance across sessions from day 1 to day 10 at both 3 and 8 months of age (Figure 7B,C). These observations were confirmed by subjecting this training data to an experience (early versus late sessions) X genotype X age analysis of variance (ANOVA). The number of errors declined with experience (F(1,42) = 56.94, p < 0.001); none of the other variables or interactions were significant.

At day 11, the same mice from the training phase were evaluated using a delayed spatial win-shift task (Figure 7D). In the initial study phase (4 arms open, 4 arms baited), the mice have free access to only 4 arms with 1 food reward per accessible arm. Upon completion of the study phase, mice experience a short retention interval of 3 minutes. In the subsequent test phase (8 arms open, 4 arms baited), mice have free access to all 8 arms, but only the 4 arms not previously visited (inaccessible during study phase) are now baited and have food rewards. During the study phase, both 3 and 8 month old mice showed no difference between groups or improvement across sessions (data not shown). This was expected, as they had already displayed high performance for this type of task by the end of the training phase.

In the test phase, 3 month old R26CT and R26CT-CRE mice show an improvement across sessions (Figure 7E) and were not different from each other. At 8 months of age, R26CT mice also showed improvement in performance across sessions (Figure 7F). In contrast, the R26CT-CRE mice did not. There was a statistically significant difference between R26CT and R26CT-CRE mice (*p < 0.05) at the end of the 10 day testing period (Figure 7F, days 18–20). These test phase data were subjected to an experience X genotype X age ANOVA. The number of errors declined with experience (F(1,42) = 28.03, p < 0.001). There was also a significant three-way interaction of days X genotype X age (F(1,42) = 8.61, p < 0.01); none of the other variables or interactions were significantly different. Next, we conducted experience X genotype ANOVAs separately for ages 3 and 8 months. At both time points, there was a significant effect of experience (3 months: F(1,21) = 17.05, p < 0.001; 8 months: (F1,21) = 11.25, p < 0.01). Moreover, the interaction of experience and genotype was significant at 8 months (F(1,21) = 14.37, p < 0.05) but not at 3 months (F(1,21) = 2.16, p = 0.16); none of the other variables were significant. These results indicate R26CT-CRE mice develop impairment in spatial working memory by 8 months of age.

### Neurophysiological evaluation

Several neurodegenerative conditions are characterized by synaptic loss or reduction in synaptic density that coincides with cognitive impairment [37]–[40]. Since 8 month old R26CT-CRE mice show cognitive impairments in spatial working memory as measured in the radial arm maze, levels of synaptophysin were measured as a qualitative indicator of synaptic density. In both 3 and 8 month old mice, levels of synaptophysin are approximately equal between R26CT and R26CT-CRE mice suggesting that spatial working memory impairments in R26CT-CRE mice are not due to decreases in synaptic density (Figures 5A-D).

**Figure 8:**
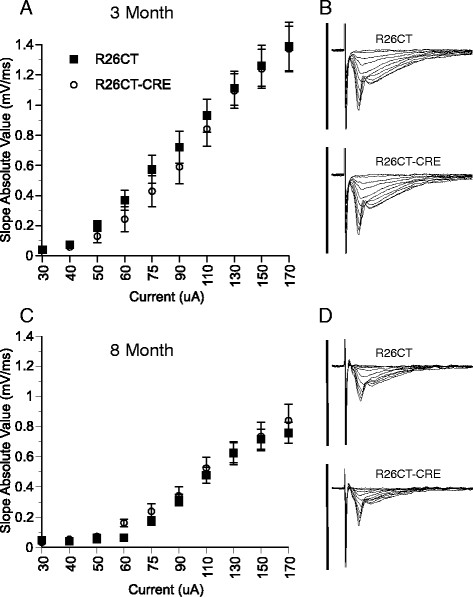
**Field excitatory post-synaptic potentials (fEPSP) recorded from the ventral hippocampus in R26CT and R26CT-CRE mice. A)** Stimulus response curves for R26CT (black squares n = 10(19)) and R26CT-CRE (open circles, n = 12(18)) mice at 3 months of age. Input intensities are 30, 40, 50, 60, 75, 90, 110, 130, 150, and 170 μA. **B)** Averaged fEPSP sweeps for each group shown in A. The vertical bar represents 2 mV and the sweeps are 50 ms in duration. **C, D)** Same as A and B above except at 8 months of age for R26CT (n = 10(20)) and R26CT-CRE (n = 12(19)). The values represent the mean ± SEM from n slices. There are no significant differences between genotypes at either 3 or 8 months of age. Significance between genotypes was determined using an unpaired t-test.

**Figure 9:**
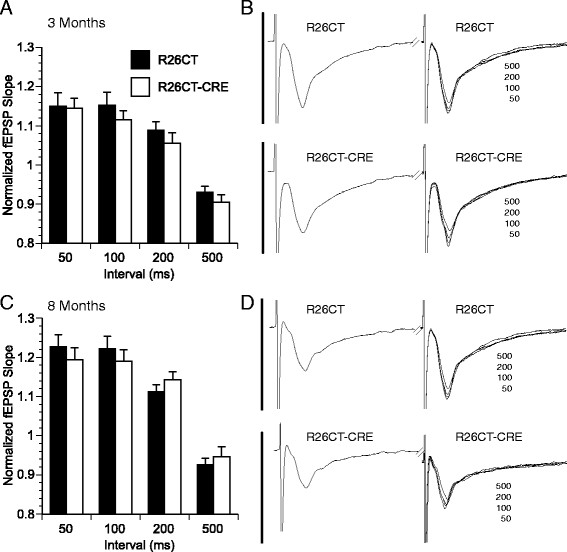
**Paired-pulse field excitatory post-synaptic potentials (fEPSP) recorded from the ventral hippocampus in R26CT and R26CT-CRE mice. A)** Paired-pulse ratios at 50, 100, 200, or 500 ms in R26CT (black bars, n = 10(19)) and R26CT-CRE (white bars, n = 12(18)) mice at 3 months of age. **B)** Averaged fEPSP sweeps for each group shown in **A**. The second sweeps for each interval are overlaid. Vertical bars represent 2 mV and sweeps are 90 ms in duration. **C, D)** Same as **A** and **B** above except at 8 months of age for R26CT (n = 10(20)) and R26CT-CRE (n = 12(21)) mice. The values represent the mean ± SEM from n slices. There is no significant difference between genotypes at either age. Significance between genotypes was determined using an unpaired t-test.

Electrophysiological studies were performed on slices obtained from both the ventral and dorsal hippocampus of each mouse. Recordings from the ventral hippocampus were performed in order to compare the physiological consequences of model Hirano bodies formed in the CA1 (this study) versus the CA3 region [28]. In order to determine whether the presence of Hirano bodies impact synaptic function, field excitatory post-synaptic potentials (fEPSPs) were recorded at the Schaffer collateral synapses in the stratum radiatum layer of the CA1 region in slices from the ventral half of the hippocampus. In 3 and 8 month old mice, there was no difference in synaptic response between R26CT (3 month: 10(19) and 8 month: 10(20)) and R26CT-CRE (3 month: 12(18) and 8 month: 12(19)) mice for fEPSPs at any stimulus intensity recorded (Figure 8). Short-term synaptic plasticity was evaluated using paired pulse stimulus protocols. There was no difference in the amount of facilitation between R26CT (3 month, n = 10(19); 8 month, n = 10(20)) and R26CT-CRE (3 month, n = 12(18); 8 month, n = 12(21)) mice at any stimulus interval at either 3 or 8 months of age (Figure 9). These results indicate that impairments in spatial working memory in 8 month R26CT-CRE mice are likely not attributed to differences in synaptic transmission or short-term plasticity in the ventral hippocampus.

**Figure 10:**
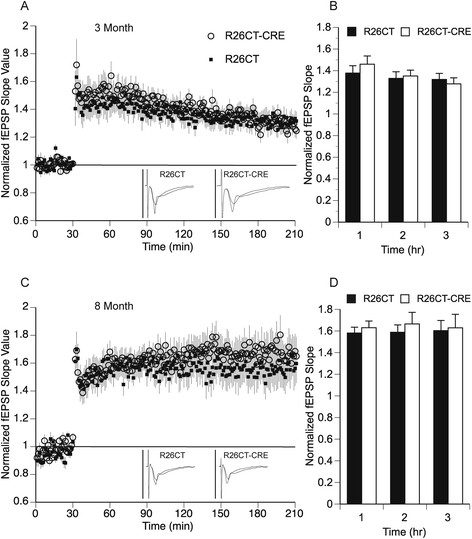
**Long-term potentiation of field excitatory post-synaptic potentials (fEPSP) recorded from the ventral hippocampus in R26CT and R26CT-CRE mice. A)** Summary time course of normalized fEPSP slope values in 3 month old R26CT mice in LTP (black square, (n = 10(17)) and R26CT-CRE (open circle, n = 11(16)), before and after high frequency stimulation (HFS) (3 x 100 Hz/1 s at 20 s intervals) indicated by the arrow at 30 minutes. Insets represent averaged fEPSP sweeps before and after HFS. The vertical bar is 2 mV. **B)** Summary quantification of LTP for R26CT and R26CT-CRE mice at 1, 2, and 3 hrs post-HFS. **C, D)** Same as panel **A** and **B** above except for 8 month old R26CT (black square, (n = 9(18)) and R26CT-CRE (open circle, (n = 11(18)). The values represent the mean ± SEM from n slices. There is no significant difference between genotypes at either age. Significance was determined using an unpaired t-test between genotypes.

Long-term synaptic plasticity was evaluated by the induction of long-term potentiation (LTP) using a strong stimulus protocol (3 × 100Hz/1 sec at 20 sec intervals). Following LTP induction, fEPSP slopes were recorded for 3 hours post-induction to measure early (1 hours) and late (3 hours) phases of LTP. At both 3 and 8 months of age, fEPSP slope values obtained from the ventral hippocampus were not significantly different between R26CT (3 month, n = 10(17); 8 month, n = 9(18)) and R26CT-CRE (3 month, n = 11(16); 8 month, n = 11(18)) mice at both early and late phases of LTP (Figure 10).

**Figure 11:**
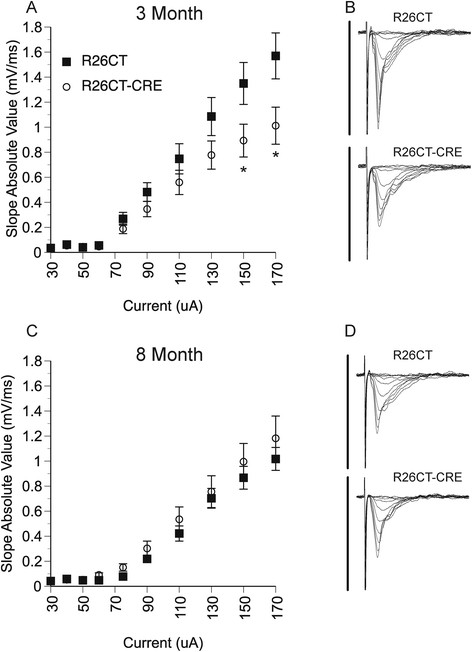
**Field excitatory post-synaptic potentials (fEPSP) recorded from the dorsal hippocampus in R26CT and R26CT-CRE mice. A)** Stimulus response curves for R26CT (black squares n = 10(10)) and R26CT-CRE (open circles, n = 12(13)) mice at 3 months of age. Input intensities are 30, 40, 50, 60, 75, 90, 110, 130, 150, and 170 μA. **B)** Averaged fEPSP sweeps for each group shown in **A**. The vertical bar represents 2 mV and the sweeps are 50 ms in duration. There is a significant difference between genotypes at the two highest input intensities (*p < 0.05). **C, D)** Same as **A** and **B** above except at 8 months of age for R26CT (n = 10(10)) and R26CT-CRE (n = 12(12)) control mice. There are no significant differences between genotypes at 8 months of age. Significance between genotypes was determined using an unpaired t-test. The values represent the mean ± SEM from n slices.

**Figure 12:**
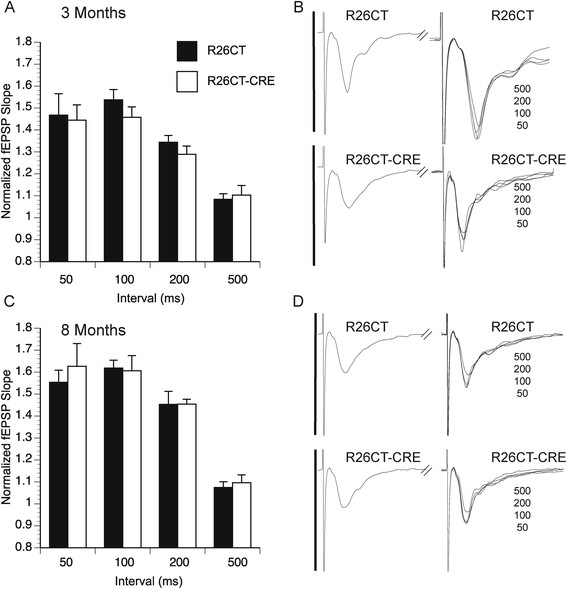
**Paired-pulse field excitatory post-synaptic potentials (fEPSP) recorded from the dorsal hippocampus in R26CT and R26CT-CRE mice. A)** Paired-pulse ratios at 50, 100, 200, or 500 ms in R26CT (black bars, n = 10(10)) and R26CT-CRE (white bars, n = 12(13)) mice at 3 months of age. **B)** Averaged fEPSP sweeps for each group shown in **A**. The second sweeps for each interval are overlaid. Vertical bars represent 3 mV and sweeps are 90 ms in duration. **C, D)** Same as **A** and **B** above except at 8 months of age for R26CT (n = 10(10)) and R26CT-CRE (n = 12(12)) mice. The values represent the mean ± SEM from n slices. There is no significant difference between genotypes at either age. Significance between genotypes was determined using an unpaired t-test.

Electrophysiology studies were also performed on slices from the dorsal hippocampus since this region has been clearly implicated in spatial memory performance. The specific role of the dorsal hippocampus in spatial working memory in particular is unclear (see Discussion). Field excitatory post-synaptic potentials (fEPSPs) were recorded at the Schaffer collateral synapses in the stratum radiatum layer of the CA1 region in slices from the dorsal half of the hippocampus as shown in Figure 11. There was a significant difference at the highest stimulus intensities tested at 3 months of age between R26CT (n = 10(10)) and R26CT-CRE (n = 12(13)) mice (*p < 0.05). In contrast, there was no difference in dorsal synaptic response between 8 month old mice R26CT (n = 10(10)) and R26CT-CRE (n = 12(12)) mice for fEPSPs at any stimulus intensity recorded. In addition, there was no difference in the magnitude of the stimulus response between the 3 and 8 month old R26CT-CRE mice, unlike the R26CT mice which declined with age. Short-term synaptic plasticity was evaluated using paired pulse stimulus protocols. There was no difference in the amount of facilitation in the dorsal hippocampus between R26CT (3 month, n = 10(10); 8 month, n = 10(10)) and R26CT-CRE (3 month, n = 12(13); 8 month, n = 12(12)) mice at any stimulus intensity at either 3 or 8 months of age (Figure 12).

**Figure 13:**
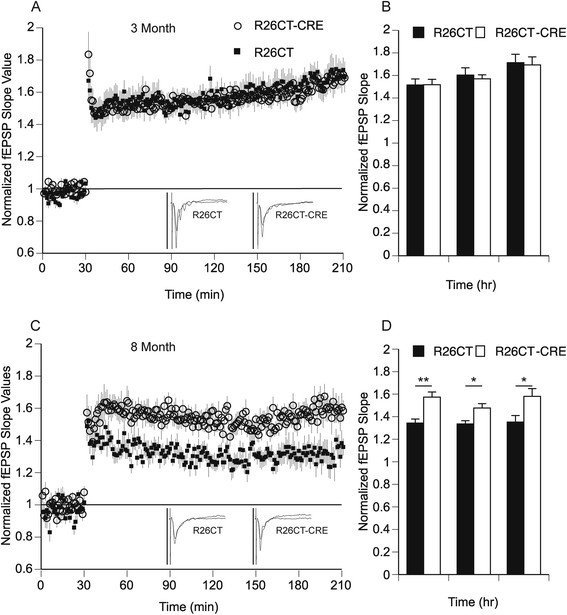
**Long-term potentiation of field excitatory post-synaptic potentials (fEPSP) recorded from the dorsal hippocampus in R26CT and R26CT-CRE mice. A)** Summary time course of normalized fEPSP slope values in 3 month old R26CT mice in LTP (black square, (n = 10(10)) and R26CT-CRE (open circle, n = 12(13)), before and after high frequency stimulation (HFS) (3 x 100 Hz/1 s at 20 s intervals) indicated by the arrow at 30 minutes. Insets represent averaged fEPSP sweeps before and after HFS. The vertical bar is 2 mV **B)** Summary quantification of LTP for R26CT and R26CT-CRE mice at 1, 2, and 3 hrs post-HFS. **C, D)** Same as panel **A** and **B** above except for 8 month old R26CT (black square, (n = 10(10)) and R26CT-CRE (open circle, (n = 12(12)). The values represent the mean ± SEM from n slices. LTP was significantly enhanced in R26CT-CRE mice at 8 months of age (*p < 0.05, **p < 0.01). Significance was determined using an unpaired t-test between genotypes.

Long-term synaptic plasticity was evaluated by the induction of long-term potentiation (LTP). Following LTP induction, fEPSP slopes were recorded for 3 hours post-induction to measure early and late phases of LTP. At 3 months of age, fEPSP slope values obtained from the dorsal hippocampus were not significantly different between R26CT (n = 10(10)) and R26CT-CRE mice (n = (12(13)) (Figure 13). However, at 8 months of age, fEPSP slope values were significantly higher for the R26CT-CRE mice (n = (10(10)) than R26CT mice (n = 12(12)), during both early (<1 hr) (**p < 0.01) and late (3 hr) (*p < 0.05) LTP. This result is unexpected given that the 8 month old R26CT-CRE mice show profound spatial working memory deficits (Figure 7F).

These results suggest that expression of CT-GFP and subsequent formation of model Hirano bodies have no significant impact on baseline synaptic transmission, short-term synaptic plasticity, or long-term synaptic plasticity in the CA1 region of the ventral hippocampus in mice as old as 8 months. In contrast, baseline synaptic transmission is significantly decreased at the highest intensities with no impact on either short-term or long-term synaptic plasticity in the dorsal CA1 region for mice presenting model Hirano bodies at 3 months of age. At 8 months of age, there is no effect on baseline synaptic transmission or short-term synaptic plasticity responses. However, there is significant enhancement of LTP that coincides with the impaired spatial working memory observed in the 8 month R26CT-CRE mice.

## Discussion

### Incidence and ultrastructure of Hirano bodies in transgenic model mice

A new transgenic mouse model was created to study the impact of Hirano bodies *in vivo* to more closely resemble the presentation of Hirano bodies in Alzheimer's disease. A mouse with model Hirano bodies had been previously studied by crossing R26CT mice with Thy1.2-CRE mice. While Hirano bodies were produced in the hippocampus of homozygous R26CT x Thy1.2-CRE mice, Hirano bodies were predominantly reported in the CA3 subregion [28]. This is not surprising since the Thy1.2-CRE mouse has been shown to have higher expression of the CRE transgene in the CA3 subregion, rather than the CA1 [28]. In the present study, R26CT mice were crossed with a CamKIIa-CRE mouse, which is known to express CRE predominantly in the forebrain and in the CA1 subregion of the hippocampus [29],[30],[41]. This CRE driver was chosen in an attempt to more closely recapitulate human disease conditions in which Hirano bodies are normally found, the pyramidal cell layer of the CA1 region [15],[16],[6],[42],[8],[12],[43]. Eosinophilic inclusions were seen predominately in the CA1 region of R26CT x CamKIIa-CRE mice. In contrast with the previously characterized model Hirano body mice (R26CT x Thy1.2-CRE) in which model Hirano bodies were not detected by light microscopy until 6 months of age, eosinophilic inclusions were found in the hippocampus of R26CT-CamKIIa-CRE mice as early as 3 months of age. It is unclear why eosinophilic inclusions are detected earlier in R26CT mice crossed with CamKIIa-CRE than in R26CT mice crossed with Thy1.2-CRE. Perhaps CA1 pyramidal neurons have a greater propensity to facilitate CT-induced Hirano body formation than do CA3 pyramidal neurons. In human disease, the number of Hirano bodies increases with age and disease severity [7],[15],[43]. Hirano body appearance in humans coincide with aging since pathologists have noted that Hirano bodies are never seen in the brains of humans younger than 11 years old [15]. Consistent with these findings, an age-dependent increase in the formation of Hirano bodies was also observed in our mouse model.

There are several characterized actin inclusions found in human disease such as ADF/cofilin rods (AC rods) and hyaline bodies [44]–[46]. Hirano bodies are differentiated from these other actin aggregates by their eosinophilic nature, ability to bind to phalloidin, and distinctive ultrastructure [47],[8],[7],[45]. To definitively prove an actin inclusion is a Hirano body, electron microscopy must be performed for ultrastructural analysis. There are a variety of presentations. In humans, they often appear as fingerprint or spheroid/spindle shape [8]. Hirano bodies in R26CT-CRE mice also display these alternative patterns (Additional file 2: Figure S2 A-B). In humans, smaller Hirano bodies observed by electron microscopy (not visible via light microscopy) are more frequent than larger Hirano bodies [16]. These smaller Hirano bodies are noted to appear less compact and more irregular than those visible through light microscopy [16]. R26CT-CRE mice also show similar smaller structures which contain both ordered filaments and amorphous electron dense material (Additional file 2: Figure S2 D). Expression of CT in cell culture systems often results in alternative fibrillar structures or small aggregates [22],[25]. These smaller structures have also been noted in the brains of humans [48] and in the brains of R26CT-CRE mice (Additional file 2: Figure S2 C). In *Dictyostelium*, small nascent model Hirano body structures observed by TEM fuse together by an unknown process involving microtubules and myosin II to form larger Hirano bodies [21],[49],[50]. It is likely that a similar formation process of Hirano bodies is occurring in R26CT-CRE mouse brains since several small aggregates can be seen in close proximity in TEM samples (Additional file 2: Figure S2 C).

### Inflammation

Inflammation is a phenomenon occurring in neurodegenerative conditions such as Alzheimer's disease and Parkinson's disease and is characterized by activated microglia and reactive astrocytes [34]. Many mouse models of neurodegenerative disease recapitulate reactive microglia or reactive astrocytes as a predominant phenotype [35],[51]–[53]. Markers of reactive astrocytes or activated microglia were utilized in order to determine if Hirano bodies accompany an innate immune response in the brain. 3 month old R26CT-CRE mice had no signs of inflammation (Figures 3, 5). However, by 8 months of age, R26CT-CRE mice had significantly higher levels of GFAP compared to age matched R26CT mice (Figures 4, 5). There are several factors that can initiate astrogliosis including cell damage, ischemia, neuronal hyperactivity, and foreign pathogens including abnormal protein aggregates [54]. In Alzheimer's disease, reactive astrocytes are often found in close proximity with deposits of amyloid beta plaques and neurons containing intracellular NFTs [35],[55]–[59]. In addition to intracellular NFTs, extracellular NFTs (ghost tangles) have also been reported in brains [60],[61]. Reactive astrocytes have been reported to surround ghost tangles where they are thought to be clearing these structures from the brain [62],[63]. Similarly, intracellular Lewy bodies comprised of alpha synuclein can be exocytosed from neurons in Parkinson's patients and initiate astrogliosis [64]–[66]. Hirano bodies have also been reported to be extruded from neurons and model Hirano bodies can be cleared from cells through exocytosis [15],[25]. It is possible that extruded Hirano bodies or intermediate aggregates may initiate an astrocytosis response in the brain. While 3 month old R26CT-CRE mice have sparse model Hirano bodies, they are more abundant in 8 month old mice. In 8 month old mice, extracellular model Hirano bodies were observed (Additional file 2: Figure S2 A). Release of model Hirano bodies may explain why 8 month old R26CT-CRE mice exhibit reactive astrocytes.

### Behavioral studies of Hirano body model mice

A central feature of this study is to assess the impact of the behavioral and physiological consequence(s) of Hirano bodies in the brain. To assess the effect of CT-GFP expression and subsequent formation of model Hirano bodies, behavioral studies were performed using both an open field test and an 8-arm radial maze. The open field test has been utilized to measure locomotor activity and anxiety [67]. At 3 and 8 months of age, R26CT and R26CT-CRE mice show similar levels of locomotor activity (Figure 6A, B). General anxiety was measured by recording the number of center zone entries between R26CT and R26CT-CRE mice (Figure 6C). No differences between genotypes at either age were found, implying that the presence of model Hirano bodies does not contribute to anxiety or impaired locomotor function.

The training phase (8 arms open, 8 arms baited) of the radial arm maze demonstrated that both R26CT and R26CT-CRE mice have intact spatial learning and navigation since both genotypes learn the reference memory rules associated with completion of the radial arm maze training task. In addition, both R26CT and R26CT-CRE mice appear to have equivalent perception of spatial cues, levels of motivation, and motor control. In the training phase, the task is continuous and thus the spatial working memory load is low, making the procedure primarily dependent upon immediately accessible information from short-term memory [68]. The same mice were subsequently tested in a 2-phase delayed spatial win-shift task (with a 3 minute retention interval delay) to more stringently examine spatial working memory. The incorporation of a time delay forces retention of trial-unique spatial information (i.e., the mice must remember which arms were visited in phase 1 in order to successfully complete phase 2, increasing the spatial working memory load). At 3 months of age, spatial working memory appears unimpaired since both R26CT and R26CT-CRE mice perform equally well and improve with training. However, at 8 months of age, the more stringent memory load resulted in discrimination of performance between the R26CT and R26CT-CRE mice. The R26CT mice showed improvement across trials, where the R26CT-CRE mice did not. The test phase results suggest that 8 month R26CT-CRE mice have impaired spatial working memory, (i.e. memory for daily, item-specific locations). This is likely a specific impairment in spatial working memory since the training phase results indicated intact acquisition of reference memory rules, making a general impairment in learning capability unlikely. Furthermore, the memory deficiencies observed at 8 months coincides with the observed increase in frequency of Hirano bodies.

### The effect of model Hirano bodies on synaptic plasticity

Assessments of spatial memory are known to depend upon intact hippocampal function [69],[70]. With respect to the specific spatial working memory task we have utilized in our assessments, it is uncertain whether the ventral or dorsal hippocampus is the predominant region primarily responsible for behavioral performance. Dorsal and ventral hippocampal lesions, fMRI data, and genetic manipulation of NMDA and AMPA receptor subunits have revealed that the ventral and dorsal hippocampus have differential roles during behavioral and memory tasks (reviewed in [71],[72]). The actin cytoskeleton plays a key role in the cellular processes and structures that memory is thought to reside in. Actin binding proteins regulate the polymerization and depolymerization of actin into dynamic, transient structures required for synaptic transmission and memory function. Mutant forms of the Dictyostelium 34 kDa actin bundling protein are gain-of-function actin bundling proteins that induce the formation of model Hirano bodies [21],[73],[50]. Expression of these proteins causes a shift in the ratio of F:G-actin in the cell [21], and slower turnover of actin filaments in the model Hirano body [50]. Thus, the presence of model Hirano bodies cause a large perturbation to the actin cytoskeleton and potentially to synaptic functions and memory. Our studies of the electrophysiological properties of the dorsal as well as the ventral hippocampus created a unique opportunity to enhance both our understanding of the role of the actin cytoskeleton in regional hippocampal function and assess the impact of Hirano bodies on spatial working memory.

### Effect of Hirano bodies in the CA3 and CA1 regions of the hippocampus

Short-term plasticity measurements in the ventral hippocampus at 3 and 8 months of age showed that paired-pulse facilitation from R26CT-CRE mice was indistinguishable from R26CT mice (Figure 10). These results are in contrast with the previous characterization of the R26CT-CRE mice with a Thy1.2 CRE driver, which exhibited significant paired-pulse depression at a 50 ms stimulus interval [28]. The most obvious explanation for this is due to differences in the expression pattern of CT-GFP between the two mouse strains. In the current study, CT-GFP expression and Hirano body formation was more predominant in the CA1 subregion of the hippocampus versus the previous mouse model that had predominantly CA3 subregion expression and subsequent Hirano body formation [28]–[30]. In both cases, field potential recordings were performed in the stratum radiatum layer of CA1 by activating the Schaffer collateral axons (which originate in CA3) and synapse on the apical dendrites of the CA1 pyramidal neurons. In the previous mouse (R26CT x Thy1.2-CRE), Hirano bodies were formed in presynaptic CA3 neurons [28]. Paired-pulse stimulation is a measurement that reflects the active transport recovery of calcium and trafficking of neurotransmitter-containing vesicles to replenish the ready releasable pool of vesicles [74]. The trafficking of these neurotransmitter-containing vesicles is modulated by the actin cytoskeleton. The paired-pulse depression seen in the R26CT x Thy1.2-CRE mice was explained as a change in vesicular trafficking due to sequestration of F-actin in model Hirano bodies [28],[75], decreasing the amount of cellular actin available for cytoskeletal functions. In the current study using R26CT x CamKIIa-CRE mice, Hirano bodies were primarily formed in the postsynaptic CA1 pyramidal neurons. Thus, the difference in the effect of Hirano bodies on paired-pulse responses between the Thy1.2 CRE and CamKIIa-CRE driver models can be readily understood. Furthermore, these results show that the effect of model Hirano bodies on synaptic function depend on the specific location (i.e. presynaptic versus postsynaptic) in which they form.

In addition to evaluating short-term synaptic plasticity, a form of long-term synaptic plasticity, LTP, which has been shown to be involved in spatial learning and memory, was also investigated [76],[77]. Long-term potentiation has both an early and a late phase. The early phase relies on redistribution and rearrangement of available synaptic proteins while the latter requires gene expression and protein synthesis to maintain changes in synaptic strength [78]. Both the early and late phases of LTP are associated with an increase in actin assembly [79]–[81]. The actin assembly associated with the late stage of LTP involves an increase in the number and size of dendritic spines and expansion of the postsynaptic density [79],[80]. Model Hirano bodies are known to shift the filamentous to monomeric actin ratio [21], increasing the proportion of cellular F-actin which may consequently impact synaptic plasticity. Therefore, LTP was monitored for 3 hours post induction to determine if the presence of model Hirano bodies could impact either the early (1 hours) or late (3 hours) phases of LTP. Interestingly, despite the important role of actin in LTP, we found that there was no measurable difference between R26CT and R26CT-CRE mice at either 3 or 8 months of age (Figure 10) in the ventral hippocampus. These results are in contrast with those observed in our earlier study in which R26CT x Thy1.2-CRE mice showed a deficit in the early, but not the late phase LTP [28]. Thus, presence of model Hirano bodies in the CA1 region of the ventral hippocampus of R26CT-CRE mice (current R26CT × CamKIIa-CRE mouse) appears to have negligible effects on synaptic plasticity as assessed by paired-pulse and LTP measurements. Once again, the presynaptic expression of model Hirano bodies in the CA3 of the Thy1.2-CRE mouse resulted in significant effects in the former report [28] that were not observed with the postsynaptic expression pattern in the CA1 of the current CAMKIIa-CRE mouse.

### Effect of Hirano bodies in the dorsal versus ventral hippocampus

Measurements of synaptic transmission and either short-term or long-term plasticity obtained from the ventral hippocampus of R26CT and R26CT-CRE mice showed no difference between the genotypes at either 3 or 8 months of age. This contrasts with the result that R26CT-CRE mice exhibited profound spatial working memory deficits at 8 months of age.

The same electrophysiological measurements were also recorded in slices obtained from the dorsal hippocampus of the same mice. In the CA1 region of dorsal hippocampal slices, there was a significant decrease in the magnitude of baseline fEPSP responses at the two highest stimulus intensities (Figure 11A) in 3 month old R26CT-CRE mice, compared to age matched controls. These 3 month R26CT-CRE mice that exhibited a reduction at high stimulus intensities, showed no spatial working memory impairments (Figure 7E) and model Hirano bodies were observed to be present in low amounts. Interestingly, in the CA1 subfield, there are no loss of synapses [82] or age related changes in the presynaptic fiber potential from the incoming Schaffer collaterals but there is a reduction in the magnitude of fEPSP in the CA1 principal cells [83] with aging. Interestingly, there was no difference between the magnitudes of fEPSP with age for the R26CT-CRE mice, in contrast to the R26CT control mice (Figure 11A,C). There were no differences in short-term plasticity measured in the dorsal hippocampus between the genotypes of mice at either age (Figure 12). There were no differences in LTP at 3 months of age (Figure 13). However, LTP was significantly enhanced in the dorsal hippocampus of 8 month old R26CT-CRE mice, when profound spatial memory impairment was also observed (Figure 7F). The relationship between the occurrence of enhanced LTP that is coincident with memory impairments in these mice at 8 months of age is surprising as most memory impairments are accompanied by decreased LTP.

Previous studies utilizing drugs affecting actin assembly have shown the importance of dynamic actin, i.e. the ability of actin to polymerize and depolymerize rapidly for maintenance of LTP but not synaptic transmission [84]–[86]. Two distinct pools of F-actin have been identified in spines: a dynamic pool with a high turnover rate of actin located near the tip of the spine and a more stable pool in the core of the spine [87],[88]. The turnover rate was estimated as 40 s and 17 min for the dynamic and stable F-actin pools, respectively [87],[89]. F-actin in model Hirano bodies formed in *Dictyostelium* with the gain-of-function 34 kDa mutant E60K are very stable in the presence of latrunculin A. The F-actin content of the model Hirano body measured by the fluorescence of rhodamine-labeled phalloidin decreased 31% after 2 hours [50]. This increased F-actin stability could cause spines to form and refashion more slowly, thus impacting memory functions.

Taken together, the effects of expression of model Hirano bodies in the presynaptic CA3 of R26CT x Thy1.2-CRE mouse [28] were readily discernable compared to the postsynaptic expression in the CA1 of the current R26 x CamKIIa-CRE mouse. 8 month old R26CT-CRE mice have an impairment in spatial working memory assessed utilizing the spatial win-shift task in the radial arm maze despite no significant differences in either short-term or long-term plasticity measurements in the ventral hippocampus that could readily explain this cognitive deficit [90],[91]. This seeming discrepancy could be due to involvement of both the hippocampus and pre-frontal cortex in spatial working memory [68],[92]. The CamKIIa promoter used to drive CRE expression is activated in the hippocampus as well as the frontal cortex [29],[30]. Thus, it is possible that the electrophysiological measurements in the CA1 of ventral hippocampus performed in the current study either do not detect changes in synaptic physiology relevant for this form of spatial working memory, or that postsynaptic localization of model Hirano Bodies does not significantly impact either paired-pulse or long-term potentiation forms of synaptic plasticity in the ventral hippocampus. In contrast, significantly enhanced LTP was observed in the dorsal hippocampus for 8 month old R26CT-CRE, coincident with spatial working memory impairments. Thus with respect to the measurements of synaptic transmission and plasticity assessed in the current study, the presence of Hirano bodies differentially affects both the CA1 and CA3 regions, as well as the dorsal and ventral sectors of the hippocampus. In the future, it will be important to perform electrophysiological recordings in the prefrontal cortex to evaluate the contribution of that brain region to spatial working memory in the 8 month old R26CT-CRE mice. In addition, it will be important to characterize the morphology of the hippocampal spines to assess the effect of perturbing the actin cytoskeleton by the presence of Hirano bodies in the CA1 region.

## Conclusion

The physiological impact of Hirano bodies in the brain has remained elusive. The transgenic mouse generated in this study provides an animal model of Hirano body formation in the mammalian brain. Consistent with humans, this transgenic mouse develops Hirano bodies in the CA1 region of the hippocampus as well as in the frontal cortex. Behavioral analyses of mice, which develop model Hirano bodies, indicate that Hirano bodies negatively impact spatial working memory. This study shows that Hirano body formation initiates an inflammatory response in the hippocampus and suggests that Hirano bodies may independently contribute to disease progression or exacerbate the disease state. This model mouse provides a tool to investigate how the presence of Hirano bodies may impact the progression of Alzheimer's disease and other neurodegenerative diseases.

## Authors' contributions

All authors in this manuscript contributed to the design of the study, analysis/interpretation of data, and drafting of this manuscript. MFurgerson, JKC, and RF carried out all experiments under the supervision of MFechheimer, RF, or JJW. MFurgerson performed the immunohistochemistry, western blot analysis, immunofluorescence, and electron microscopy. JKC performed the electrophysiology experiments, open field measurements and analysis. MFurgerson, RF, and JKC performed the radial arm maze experiments. JDC provided and designed the radial arm maze protocol as well as performed statistical analysis of the radial arm maze data. All authors have read and approved this manuscript for publication.

## Additional files

## Electronic supplementary material

Additional file 1: Figure S1.: Immunofluorescence and western blot of CT-GFP expression in R26CT-CRE mice. A) Immunofluorescence microscopy was performed on cryosections from 1 month old R26CT and R26CT-CRE mouse brains. Sections were stained with anti-GFP antibodies to visualize expression of CT-GFP and counterstained with DAPI and TRITC-labeled phalloidin to visualize nuclei and F-actin, respectively. R26CT-CRE mice show expression of CT-GFP in the hippocampus while R26CT control mice do not. B) A western blot was performed using brain homogenate from hippocampus and cortex of 1 month old R26CT and R26CT-CRE mice using anti-GFP antibodies to detect CT-GFP expression. To ensure no expression of CT-GFP is detectable in R26CT mice, twice the amount of protein from R26CT samples was loaded compared to R26CT-CRE samples. R26CT-CRE mice show strong expression of CT-GFP in the hippocampus and weak expression in the cortex. R26CT mice have no detectable CT-GFP in either hippocampus or cortex. Scale bar represents 40 or 200 μm. (PNG 2 MB)

Additional file 2: Figure S2.: Electron micrographs of inclusions in 8 month R26CT-CRE mice. Hippocampal tissue from 8 month old R26CT and R26CT-CRE mice was isolated and prepared for transmission electron microscopy. R26CT-CRE tissue contained electron dense inclusions which are identical to the ultrastructure of Hirano bodies. These structures were not observed in R26CT mice (data not shown). A, B) The ultrastructure of model Hirano bodies resembling a spheroid or fingerprint pattern similar to those seen in humans [8]. C) Intermediate structures were seen in the brains of R26CT-CRE mice similar to those seen in humans and cell culture models [22],[25],[48]. D) R26CT-CRE mice exhibit model Hirano bodies which contain both ordered filaments and amorphous electron dense material. Arrows indicate Hirano bodies or intermediates magnified in the panels to the right. Scale bars are in nm. (PNG 6 MB)

Below are the links to the authors’ original submitted files for images.

Authors’ original file for figure 1

Authors’ original file for figure 2

Authors’ original file for figure 3

Authors’ original file for figure 4

Authors’ original file for figure 5

Authors’ original file for figure 6

Authors’ original file for figure 7

Authors’ original file for figure 8

Authors’ original file for figure 9

Authors’ original file for figure 10

Authors’ original file for figure 11

Authors’ original file for figure 12

Authors’ original file for figure 13
