# Supplementary figures and images for: Hirano body expression impairs spatial working memory in a novel mouse model
Source: Acta Neuropathol Commun. 2014 Sep 2;2:131. doi: 10.1186/s40478-014-0131-9 (PMC4160558; doi:10.1186/s40478-014-0131-9)

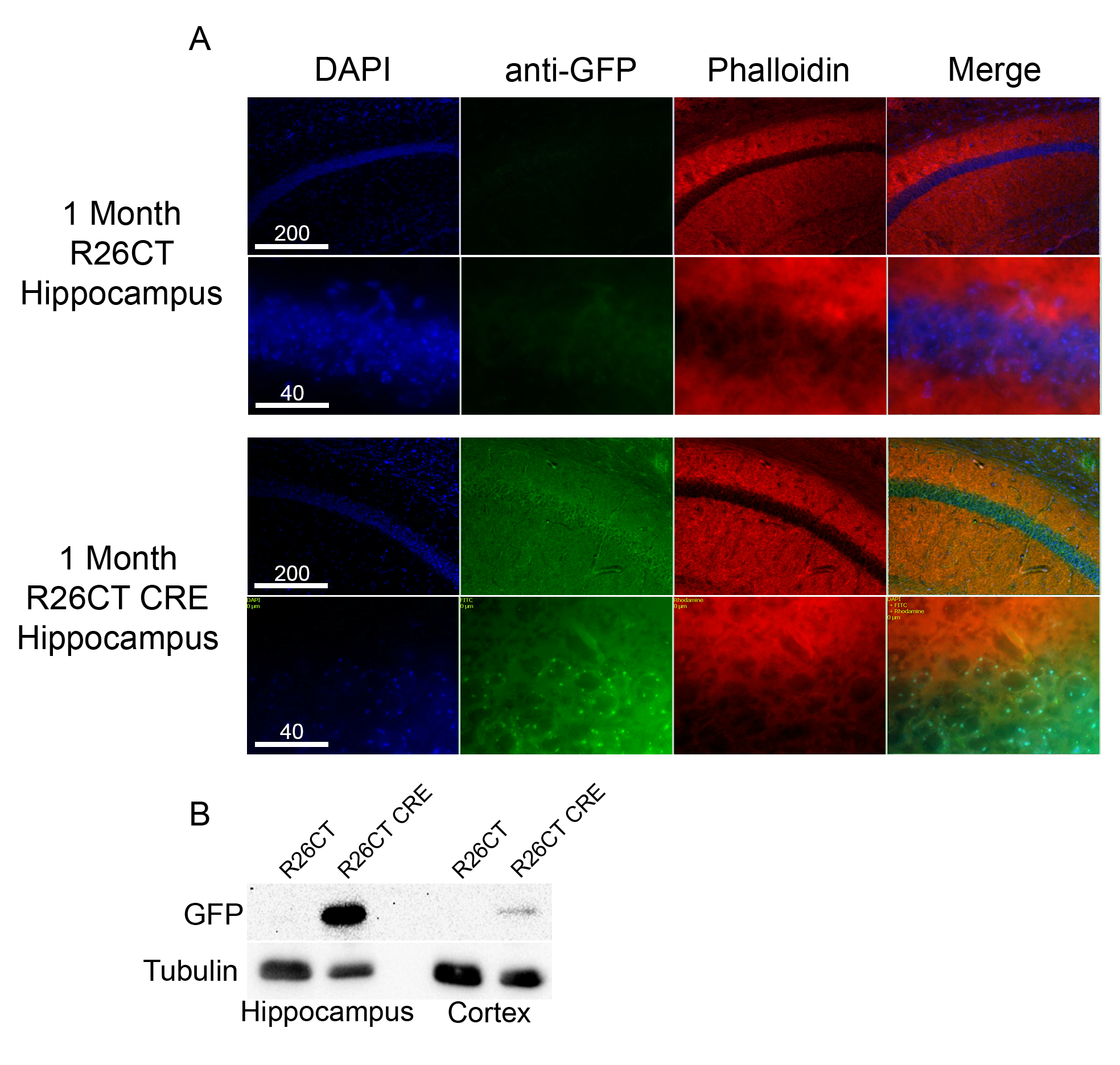

Supplement: Supplementary file 1 — Additional file 1: Figure S1.: Immunofluorescence and western blot of CT-GFP expression in R26CT-CRE mice. A) Immunofluorescence microscopy was performed on cryosections from 1 month old R26CT and R26CT-CRE mouse brains. Sections were stained with anti-GFP antibodies to visualize expression of CT-GFP and counterstained with DAPI and TRITC-labeled phalloidin to visualize nuclei and F-actin, respectively. R26CT-CRE mice show expression of CT-GFP in the hippocampus while R26CT control mice do not. B) A western blot was performed using brain homogenate from hippocampus and cortex of 1 month old R26CT and R26CT-CRE mice using anti-GFP antibodies to detect CT-GFP expression. To ensure no expression of CT-GFP is detectable in R26CT mice, twice the amount of protein from R26CT samples was loaded compared to R26CT-CRE samples. R26CT-CRE mice show strong expression of CT-GFP in the hippocampus and weak expression in the cortex. R26CT mice have no detectable CT-GFP in either hippocampus or cortex. Scale bar represents 40 or 200 μm. (PNG 2 MB) [file 40478_2014_9131_MOESM1_ESM.png]

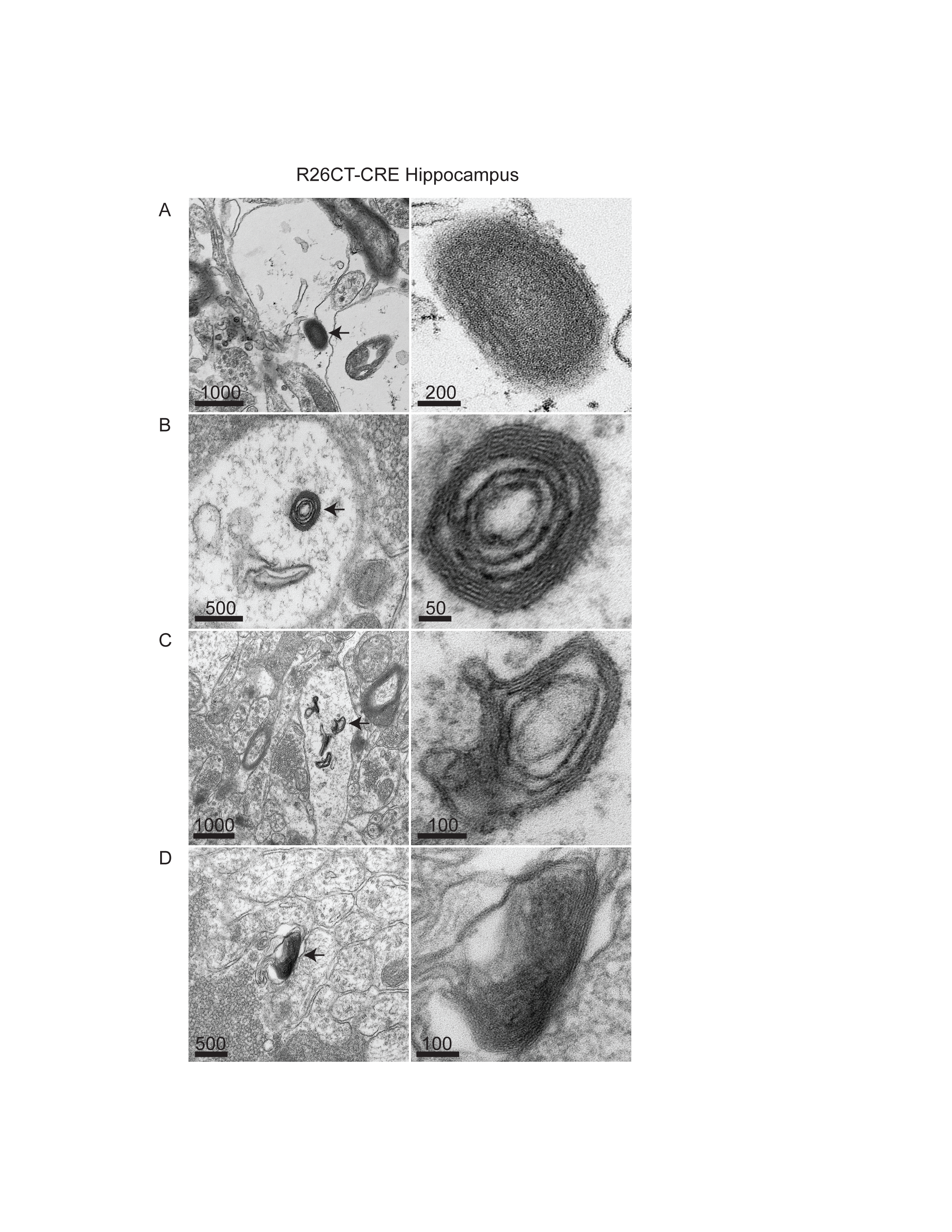

Supplement: Supplementary file 2 — Additional file 2: Figure S2.: Electron micrographs of inclusions in 8 month R26CT-CRE mice. Hippocampal tissue from 8 month old R26CT and R26CT-CRE mice was isolated and prepared for transmission electron microscopy. R26CT-CRE tissue contained electron dense inclusions which are identical to the ultrastructure of Hirano bodies. These structures were not observed in R26CT mice (data not shown). A, B) The ultrastructure of model Hirano bodies resembling a spheroid or fingerprint pattern similar to those seen in humans [8]. C) Intermediate structures were seen in the brains of R26CT-CRE mice similar to those seen in humans and cell culture models [22],[25],[48]. D) R26CT-CRE mice exhibit model Hirano bodies which contain both ordered filaments and amorphous electron dense material. Arrows indicate Hirano bodies or intermediates magnified in the panels to the right. Scale bars are in nm. (PNG 6 MB) [file 40478_2014_9131_MOESM2_ESM.png]

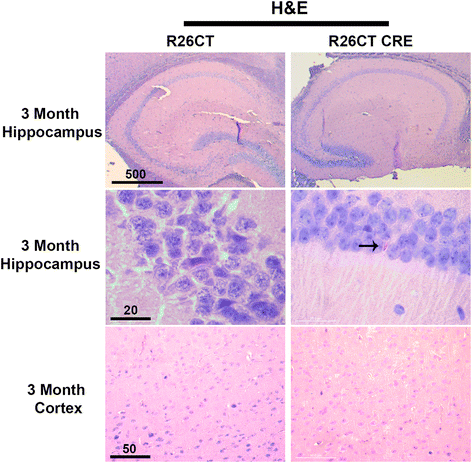

Supplement: Supplementary file 3 — Authors’ original file for figure 1 [file 40478_2014_9131_MOESM3_ESM.gif]

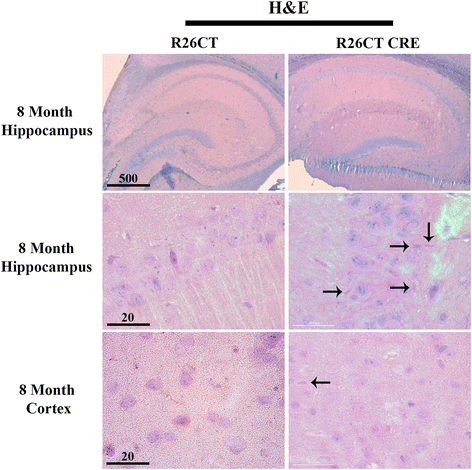

Supplement: Supplementary file 4 — Authors’ original file for figure 2 [file 40478_2014_9131_MOESM4_ESM.gif]

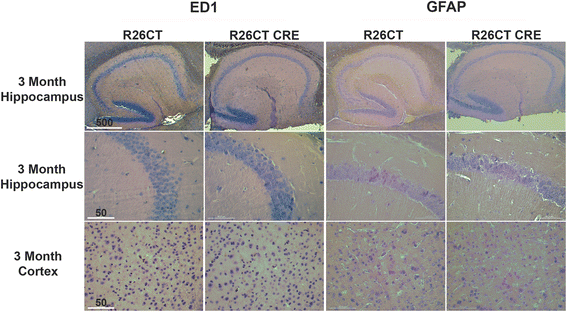

Supplement: Supplementary file 5 — Authors’ original file for figure 3 [file 40478_2014_9131_MOESM5_ESM.gif]

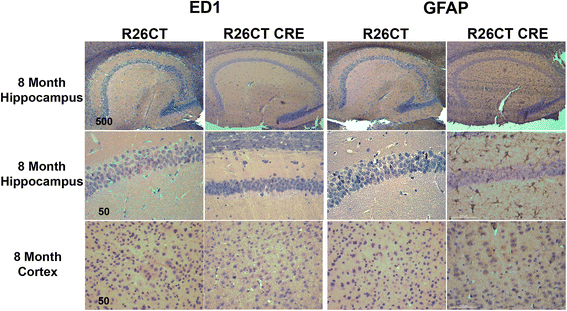

Supplement: Supplementary file 6 — Authors’ original file for figure 4 [file 40478_2014_9131_MOESM6_ESM.gif]

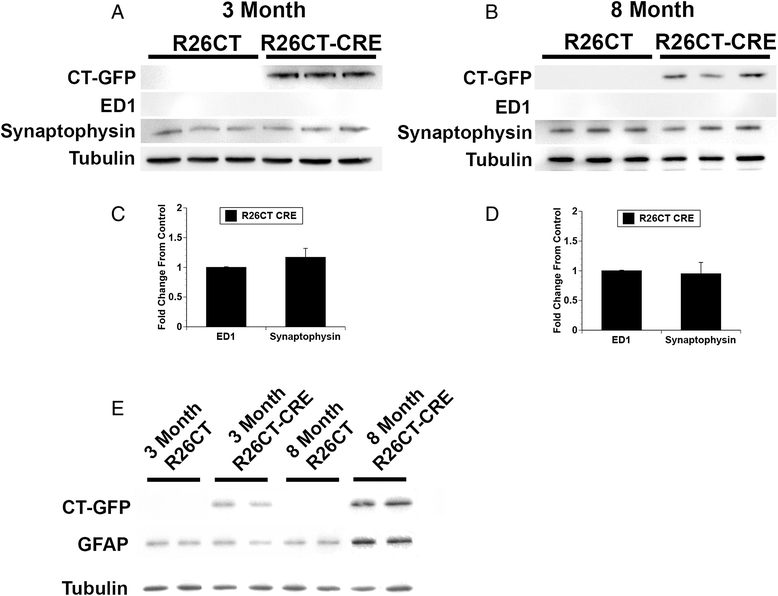

Supplement: Supplementary file 7 — Authors’ original file for figure 5 [file 40478_2014_9131_MOESM7_ESM.gif]

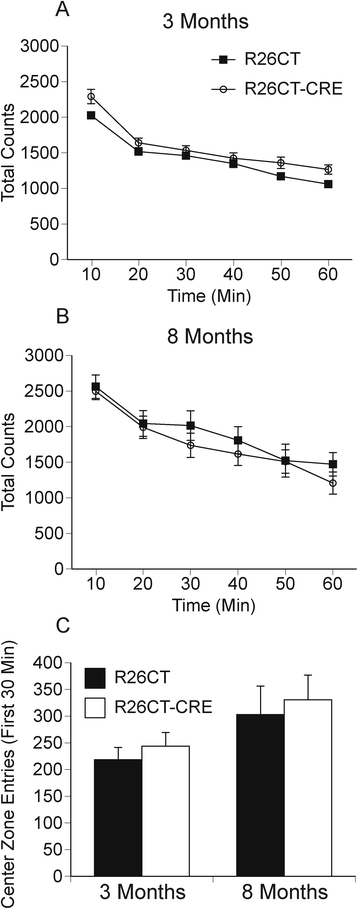

Supplement: Supplementary file 8 — Authors’ original file for figure 6 [file 40478_2014_9131_MOESM8_ESM.gif]

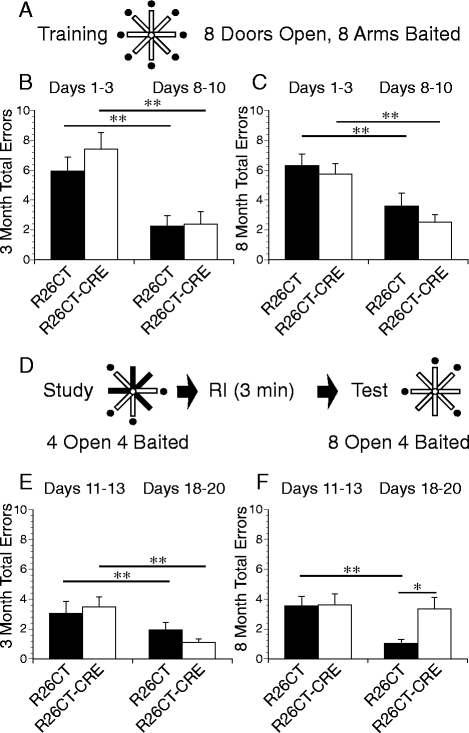

Supplement: Supplementary file 9 — Authors’ original file for figure 7 [file 40478_2014_9131_MOESM9_ESM.gif]

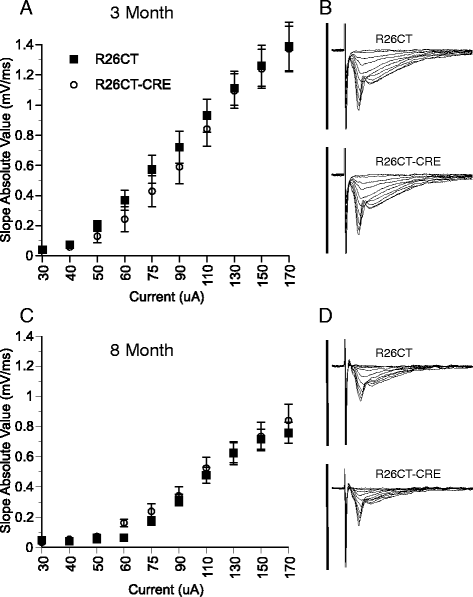

Supplement: Supplementary file 10 — Authors’ original file for figure 8 [file 40478_2014_9131_MOESM10_ESM.gif]

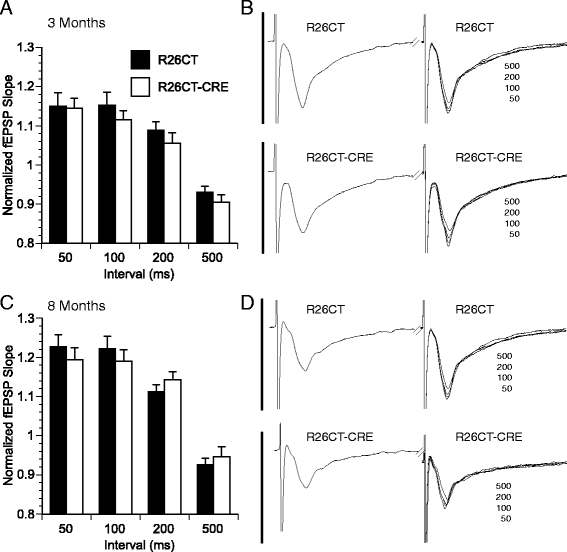

Supplement: Supplementary file 11 — Authors’ original file for figure 9 [file 40478_2014_9131_MOESM11_ESM.gif]

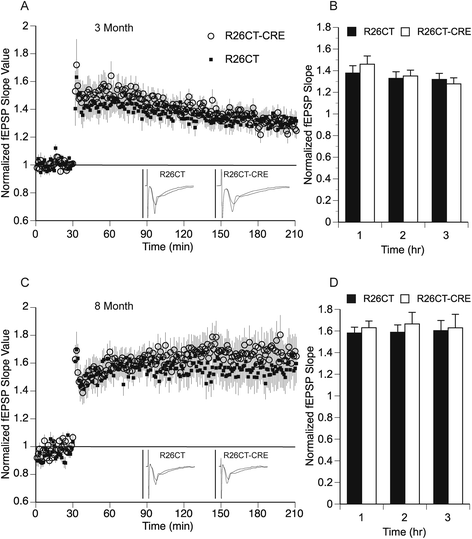

Supplement: Supplementary file 12 — Authors’ original file for figure 10 [file 40478_2014_9131_MOESM12_ESM.gif]

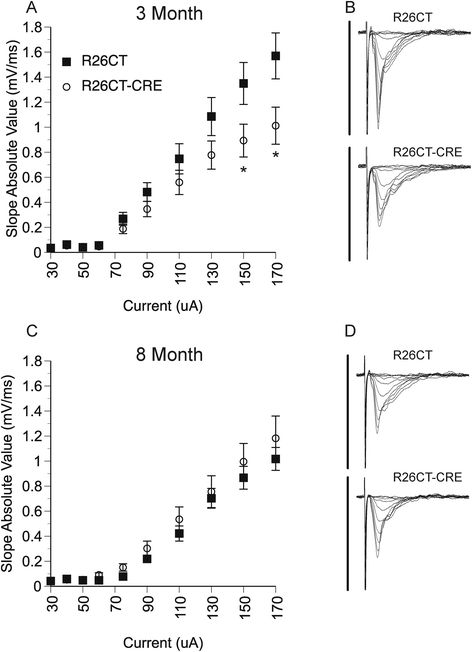

Supplement: Supplementary file 13 — Authors’ original file for figure 11 [file 40478_2014_9131_MOESM13_ESM.gif]

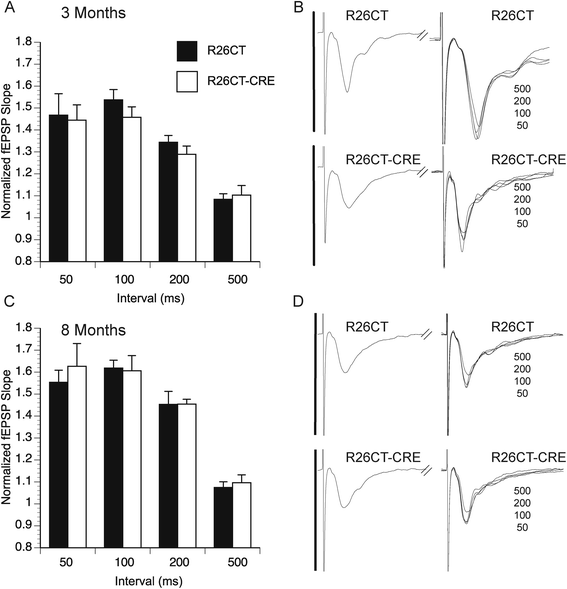

Supplement: Supplementary file 14 — Authors’ original file for figure 12 [file 40478_2014_9131_MOESM14_ESM.gif]

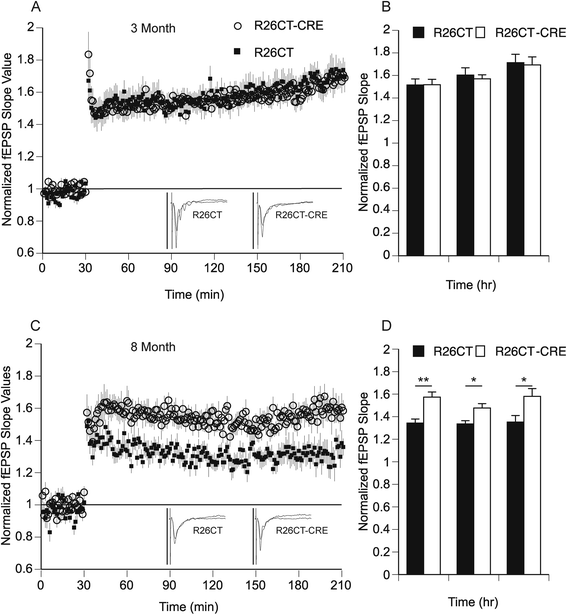

Supplement: Supplementary file 15 — Authors’ original file for figure 13 [file 40478_2014_9131_MOESM15_ESM.gif]
